# Mechanosensitive Hydrolysis of ATP and ADP in Lamina Propria of the Murine Bladder by Membrane-Bound and Soluble Nucleotidases

**DOI:** 10.3389/fphys.2022.918100

**Published:** 2022-06-16

**Authors:** Mafalda S. L. Aresta Branco, Alejandro Gutierrez Cruz, Jacob Dayton, Brian A. Perrino, Violeta N. Mutafova-Yambolieva

**Affiliations:** Department of Physiology and Cell Biology, University of Nevada Reno School of Medicine, Reno, NV, United States

**Keywords:** urothelium, bladder, nucleotidases, purine nucleotides, ATP-adenosine triphosphate, ADP, lamina propria (LP), ATP hydrolysis

## Abstract

Prior studies suggest that urothelium-released adenosine 5′-triphosphate (ATP) has a prominent role in bladder mechanotransduction. Urothelial ATP regulates the micturition cycle through activation of purinergic receptors that are expressed in many cell types in the lamina propria (LP), including afferent neurons, and might also be important for direct mechanosensitive signaling between urothelium and detrusor. The excitatory action of ATP is terminated by enzymatic hydrolysis, which subsequently produces bioactive metabolites. We examined possible mechanosensitive mechanisms of ATP hydrolysis in the LP by determining the degradation of 1,*N* ^*6*^-etheno-ATP (eATP) at the anti-luminal side of nondistended (empty) or distended (full) murine (C57BL/6J) detrusor-free bladder model, using HPLC. The hydrolysis of eATP and eADP was greater in contact with LP of distended than of nondistended bladders whereas the hydrolysis of eAMP remained unchanged during filling, suggesting that some steps of eATP hydrolysis in the LP are mechanosensitive. eATP and eADP were also catabolized in extraluminal solutions (ELS) that were in contact with the LP of detrusor-free bladders, but removed from the organ chambers prior to addition of substrate. The degradation of both purines was greater in ELS from distended than from nondistended preparations, suggesting the presence of mechanosensitive release of soluble nucleotidases in the LP. The released enzyme activities were affected differently by Ca^2+^ and Mg^2+^. The common nucleotidase inhibitors ARL67156, POM-1, PSB06126, and ENPP1 Inhibitor C, but not the alkaline phosphatase inhibitor (-)-p-bromotetramisole oxalate, inhibited the enzymes released during bladder distention. Membrane-bound nucleotidases were identified in tissue homogenates and in concentrated ELS from distended preparations by Wes immunodetection. The relative distribution of nucleotidases was ENTPD1 >> ENPP1 > ENTPD2 = ENTPD3 > ENPP3 = NT5E >> ENTPD8 = TNAP in urothelium and ENTPD1 >> ENTPD3 >> ENPP3 > ENPP1 = ENTPD2 = NT5E >> ENTPD8 = TNAP in concentrated ELS, suggesting that regulated ectodomain shedding of membrane-bound nucleotidases possibly occurs in the LP during bladder filling. Mechanosensitive degradation of ATP and ADP by membrane-bound and soluble nucleotidases in the LP diminishes the availability of excitatory purines in the LP at the end of bladder filling. This might be a safeguard mechanism to prevent over-excitability of the bladder. Proper proportions of excitatory and inhibitory purines in the bladder wall are determined by distention-associated purine release and purine metabolism.

## 1 Introduction

The urinary bladder has two main functions: storage (continence) and voiding (micturition) of urine. Normal operation of these functions depends on proper detrusor excitability regulated by systemic (e.g., *via* the central and peripheral nervous systems) and local (e.g., *via* urothelial mediators and signal propagation in cell syncytium) mechanisms. While neural control of the bladder has been extensively investigated, local mechanisms of mechanotransduction from mucosa (urothelium) to nerves or detrusor during bladder filling have remained elusive (Dalghi et al., 2020). Adenosine 5′-triphosphate (ATP) has attracted much attention in urothelium biology after the seminal discovery in 1997 that ATP was released from rabbit bladder mucosa sheets mounted in Ussing chambers in response to hydrostatic pressure (Ferguson et al., 1997). In the following decades, studies have confirmed that ATP is released from bladder sheets of different species (Wang et al., 2005; Lewis and Lewis, 2006; Yu, 2015); from cultured urothelial cells upon hydrostatic pressure changes, stretch, hypotonicity-induced cell swelling, or drag forces (Matsumoto-Miyai et al., 2011; McLatchie and Fry, 2015); and, in bladder lumen at end of filling (Vlaskovska et al., 2001; Beckel et al., 2015; Durnin et al., 2016). We recently demonstrated that during bladder filling, ATP is not only released into the bladder lumen, but it is also released from the urothelium into the suburothelium/lamina propria (LP) (Durnin et al., 2019b). This finding provided more direct support to the assumption that ATP may be released in the LP to transmit information to the nervous and muscular systems during bladder filling (Birder and Andersson, 2013; Takezawa et al., 2016b). ATP increases the tone of the detrusor *via* stimulation of P2X and P2Y purinergic receptors on smooth muscle cells (Burnstock, 2014) and is proposed to activate the micturition reflex *via* stimulation of purinergic receptors in afferent neurons in the LP at the end of filling (Cockayne et al., 2000; Vlaskovska et al., 2001). Studies have suggested that release of ATP from the bladder urothelium might be enhanced in disease states such as inflammation, forms of overactive bladder, painful bladder syndrome, and cancer (Sun and Chai, 2006; Kumar et al., 2007; Silva-Ramos et al., 2013; Burnstock, 2014). Of particular importance is the finding that in addition to ATP, its metabolites adenosine 5′-diphosphate (ADP), adenosine 5′-monophosphate (AMP), and adenosine (ADO) are physiologically present in the LP during bladder filling (Durnin et al., 2019b). It is noteworthy to point out that ATP represents only ∼5% of the purine pool available deep in the bladder wall at the end of bladder filling (Durnin et al., 2019b), suggesting that a significant degradation of ATP likely occurs in the LP during bladder filling. This would diminish the active concentrations of ATP in LP limiting the activation of P2X_2_/X_3_ receptors in afferent neurons and initiation of a voiding reflex. Therefore, understanding the mechanisms of ATP hydrolysis in the course of bladder filling becomes critically important for comprehending mechanotransduction mechanisms in the bladder wall. The study of purinergic receptors and their functions in regulation of bladder excitability is complicated by the presence at the cell surface of many enzymes—nucleotidases—that catabolize purine nucleotides into nucleosides. While hydrolysis of extracellular ATP terminates its direct action, it also generates ADP and ADO, both of which could affect bladder excitability. ADP is a potent agonist of P2Y_1_, P2Y_12_, and P2Y_13_ purinergic receptors (Abbracchio et al., 2006) and stimulation of the P2Y_12_ receptor results in detrusor contractions (Yu et al., 2014). ADO is a ligand for four ubiquitous G-protein coupled receptors (A1, A2A, A2B, and A3) (Fredholm et al., 2000), relaxes the bladder detrusor (Burnstock, 2014) and regulates the activity of sensory neurons in the bladder wall (Kitta et al., 2014). An effect thought to be due to ATP may, in fact, involve its hydrolysis products ADP or ADO. Significant amount of knowledge about extracellular nucleotide metabolism in numerous systems has been accumulated in the past 20 years (Zimmermann et al., 2012; Zimmermann, 2021). However, the information about degradation of extracellular purines in the urinary bladder is surprisingly sparse (Lewis and Lewis, 2006; Yu et al., 2011; Yu, 2015).

**FIGURE 1:**
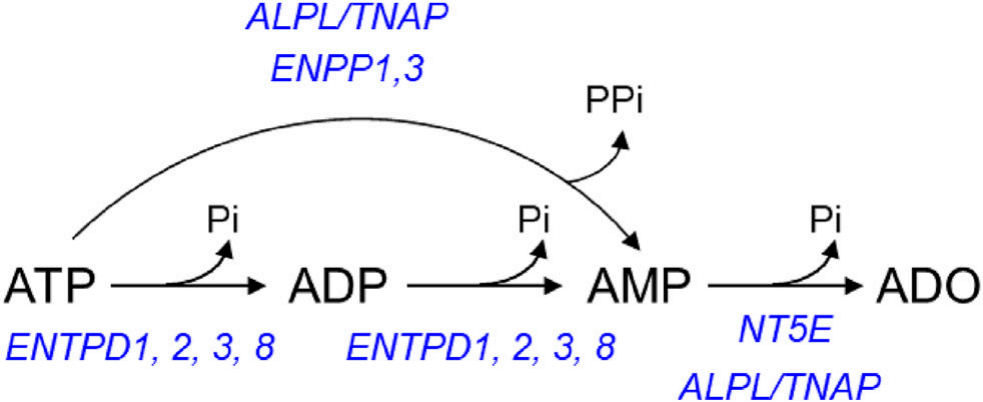
Principal pathways of extracellular ATP hydrolysis. ATP is hydrolyzed to ADP and then ADP is hydrolyzed to AMP by ENTPD1, 2, 3, and 8. ATP can be degraded directly to AMP by alkaline phosphatase (ALPL/TNAP) and ENPP1 and 3. AMP, in turn, is degraded to ADO by NT5E and ALPL/TNAP.

The extracellular metabolism of ATP is remarkably complex (Zimmermann et al., 2012): ATP is degraded sequentially to ADP, AMP, and ADO in the extracellular space by ectonucleoside triphosphate diphosphohydrolases (ENTPDs), ecto-nucleotide pyrophosphatase/phosphodiesterases (ENPPs), alkaline phosphatase/tissue-nonspecific isozyme (TNAP), and 5′-nucleotidase (NT5E/CD73) (Figure 1). The contribution of nucleotidases in the degradation of urothelial ATP might be highly specialized (Yu et al., 2011; Yu, 2015). While release of ATP by mechanical stimulation of bladder mucosa or cultured urothelial cells has been reported, it is currently unknown whether mechanical stretch during bladder filling affects the degradation of ATP in the LP. Understanding of these mechanisms is important for comprehending the regulation of bladder excitability by excitatory and inhibitory purine mediators during different stages of bladder filling.

The present study was designed, therefore, to evaluate 1) how distention of the bladder wall during bladder filling affects the hydrolysis of ATP and other purines in the LP and 2) the possible involvement of membrane-bound and soluble enzymes in these processes.

## 2 Materials and Methods

### 2.1 Animals

Adult (11–16 weeks) C57BL/6J male and female mice (Jackson Laboratory, Bar Harbor, MN) were used in this study. Mice were euthanized by sedation with isoflurane followed by cervical dislocation and exsanguination. The urinary bladder was removed and placed in oxygenated ice-cold Krebs-bicarbonate solution (KBS) with the following composition (mM): 118.5 NaCl, 4.2 KCl, 1.2 MgCl_2_, 23.8 NaHCO_3_, 1.2 KH_2_PO_4_, 11.0 dextrose, and 1.8 CaCl_2_ (pH 7.4) and subject to further dissection.

### 2.2 Ethical Approval

Animals were maintained and experiments were performed in accordance with the National Institutes of Health Guide for the Care and Use of Laboratory Animals and the Institutional Animal Use and Care Committee at the University of Nevada.

### 2.3 *Ex vivo* Detrusor-Free Bladder Preparation

*Ex vivo* bladder preparations with detrusor smooth muscle removed were prepared as described previously (Durnin et al., 2019a; Durnin et al., 2019b). Briefly, the bladder was pinned to a Sylgard-covered dissecting dish filled with cold oxygenated KBS by the urethra, ureters, and bladder serosa at the apex. After cleaning the fat and connective tissue that surrounds the bladder and ureters, portions of the serosa with the muscle attached were pulled gently and cut with fine surgical scissors along the submucosal surface of the muscle layer without touching the urothelium. Once all the detrusor was removed, a PE-20 catheter was placed through the urethra and secured with 6-0 silk and 6-0 nylon sutures.

### 2.4 Evaluation of Nucleotidase Activities in Suburothelium/Lamina Propria of Detrusor-Free Bladder Preparations

#### 2.4.1 General Protocol of Enzymatic Reactions

1,*N* ^6^-etheno-derivatives of ATP (eATP), ADP (eADP), and AMP (eAMP) (2 µM each) were used as substrates. Reactions were performed at 37°C either in the presence of bladder preparations or in solutions collected from baths that previously contained bladder preparations (called extraluminal solutions, ELS). Following addition of substrate (0 min), 20 µl samples were collected from the reaction solution at 10 s, 2, 4, 6, 8, 10, 20, 30, 40, and 60 min and diluted 10-fold in ice-cold citric phosphate buffer (pH 4.0) to stop the enzymatic reactions. Collected samples were compared with a 2 µM substrate in KBS that has not been in contact with enzyme (designated as “beaker” sample). All samples were stored at −20°C until final analysis. Substrate decrease and product increase was evaluated by ultrasensitive HPLC-FLD methodology as described in Section 2.8 HPLC analysis of 1,*N* ^*6*^-etheno-nucleotides. Each e-purine was expressed as percent of total purines detected in each sample. Only one substrate was used in each bladder preparation.

#### 2.4.2 Nucleotidase Activity in Lamina Propria in the Presence of Bladder Preparation (Protocol 1)

**FIGURE 2:**
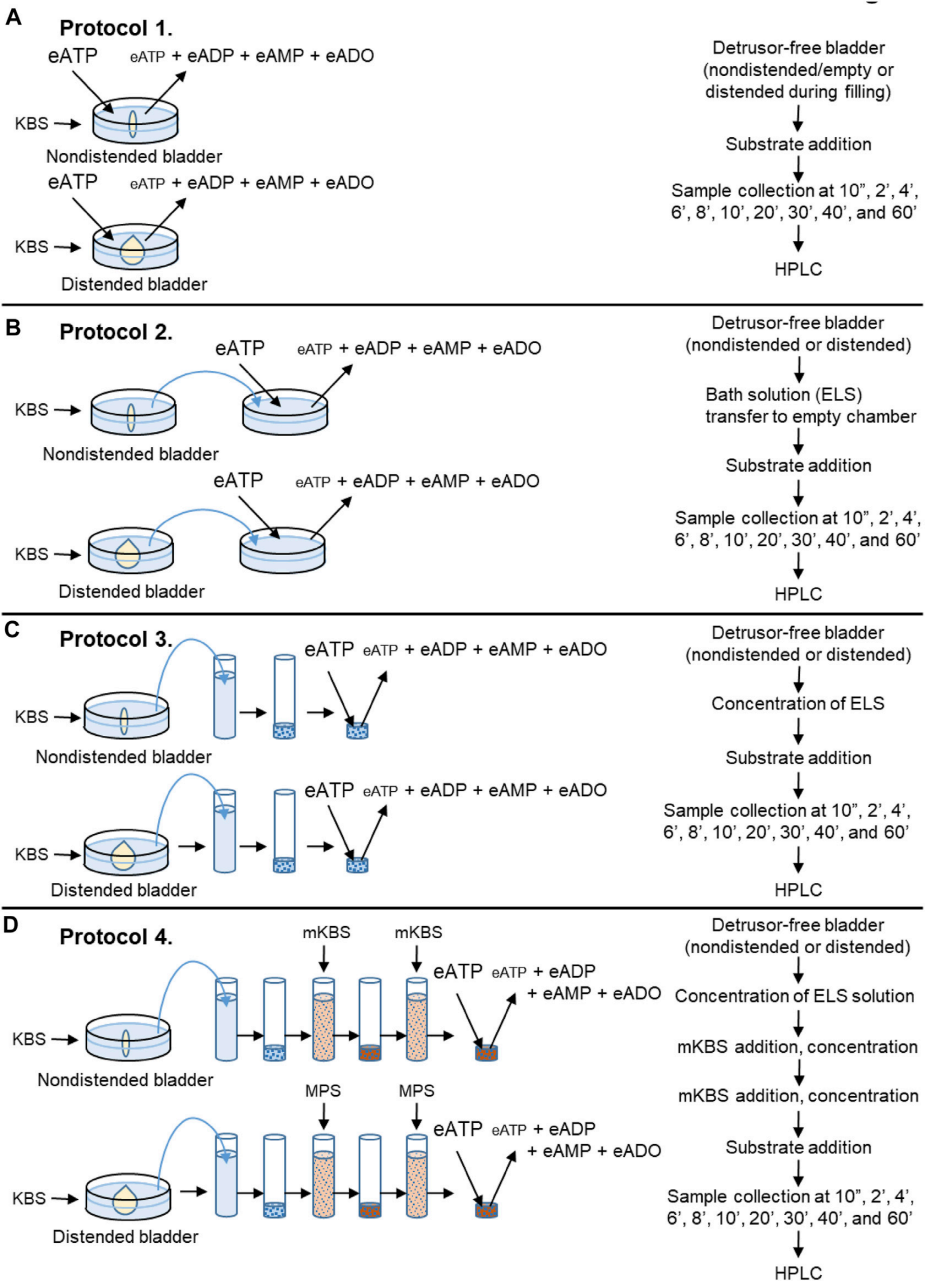
Schematics of experimental procedures for evaluation of eATP degradation in extraluminal solutions from nondistended and distended detrusor-free bladder preparations. **(A)** Substrate (e.g., eATP or eADP) was added to KBS bathing the bladder preparation. Decrease of substrate and increase of product was measured using HPLC-FLD. **(B)** Bladder was incubated in KBS. After incubation time equivalent to the time for bladder filling, an aliquot of the bath solution (aka extraluminal solution, ELS) was transferred to an empty chamber. Substrate (e.g., eATP or eADP) was added to the transferred ELS and substrate hydrolysis was assessed by HPLC. **(C)** Bladder was incubated in KBS as described in panel **(B)**. Then an aliquot of ELS was placed in a filtration unit (MWCO 10 kDa) and centrifuged. The supernatant (aka concentrated ELS, cELS) was transferred to an Eppendorf tube to which the substrate was added and substrate decrease and product increase were evaluated using HPLC. **(D)** Bladder was incubated with KBS. Then, an aliquot of ELS was concentrated as described in panel **(C)**. Next, 2.9-ml of modified KBS (mKBS) (Table 1) was added in the centrifugal unit with EL supernatant in KBS and centrifuged. Then, 2.9-ml of mKBS was added to the centrifugal unit containing the EL supernatant in mKBS and centrifuged. The resulting supernatant was transferred to an Eppendorf tube and the volume of cELS was brought to 200 µl with mKBS (37°C). Substrate was added to the Eppendorf tube and substrate decrease and product increase was measured by HPLC.

Detrusor-free bladder preparations were placed in 3-ml water jacketed chambers filled with KBS at 37°C, bubbled with 95% O_2_/5% CO_2_. After equilibration for 20 min, KBS in the chamber was replaced with fresh KBS and the bladder was either left empty (nondistended condition) for the time equivalent to filling or filled at 15 μl/min with KBS *via* an infusion pump (Kent Scientific, Torrington, CT) to ∼85–90% of bladder capacity determined at time of dissection (distended condition). Such filling volumes were similar to the volumes that were necessary to generate pre-voiding intravesical pressure (Durnin et al., 2019b), and were considered pre-voiding volumes. The degradation of eATP, eADP or eAMP was evaluated in the chamber containing 2.5 ml substrate solution in contact with nondistended or distended bladder preparations (Figure 2A, Protocol 1).

#### 2.4.3 Soluble/Releasable Nucleotidase Activity in Lamina Propria (Protocols 2 and 3)

Following equilibration, the KBS in the chamber containing a bladder preparation was replaced with 3 ml of fresh KBS and the bladder was either left empty for the time equivalent to filling or filled at 15 μl/min with KBS to pre-voiding volume. Then, 2.4 ml of bathing solution (i.e., ELS) that was in contact with the bladder preparation was transferred to an empty water-jacketed chamber with an oxygen line. Substrate eATP, eADP or eAMP was added to the transferred solution at a final concentration of 2 µM and its degradation in the absence of tissue was evaluated for 1 h (Figure 2B, Protocol 2).

In a separate set of experiments, 2.9-ml of ELS was collected from nondistended or distended bladders and concentrated using 4-ml Amicon Ultra centrifugal filter units with a 10 kDa molecular weight cut-off pore size (Millipore Sigma, Burlington, MA, United States). Samples were concentrated by centrifugation at 4,000 × g for 25 min at 4°C using swing bucket rotor (ThermoFisher Scientific SorvallST 40R, Langenselbold, Germany). The concentrated EL solutions (cELS) were brought to 200 µl with oxygenated KBS and enzymatic reactions were performed at 37°C by adding substrate eATP, eADP or eAMP to the cEL sample (Figure 2C, Protocol 3).

#### 2.4.4 Effects of Extracellular Ca^2+^ and Mg^2+^ on the Enzymatic Activities of Soluble (Released) Nucleotidases (Protocol 4)

Denuded bladder preparations were placed in water jacketed chambers filled with oxygenated KBS at 37°C. Following the equilibration, the KBS in the chamber was replaced with fresh KBS and the bladder was either left nondistended for the time equivalent to filling or it was filled (distended) at 15 μl/min with KBS to pre-voiding filling volume. This step ensured that soluble enzymes, if available, were released in regular KBS. Then, 2.9 ml of EL solution from nondistended or distended preparation was concentrated to approximately 80 µl using Amicon Ultra centrifugal filter units (10 kDa MWCO, 4-ml, and 25 min) as described in Section 2.4.3 Soluble/Releasable Nucleotidase Activity in Lamina Propria (Protocol 3). In the next step, 2.9 ml of modified KBS (mKBS) that lacked Ca^2+^ and/or Mg^2+^ in the absence or presence of 5 mM EGTA and/or 5 mM EDTA (Table 1) was added to the centrifugal filter unit with cELS in KBS and was centrifuged at 4,000 × g for 15 min. To ensure complete replacement with mKBS, another 2.9-ml of mKBS was added to the centrifugal unit and concentrated at 4,000 × g for 25 min (Figure 2D, Protocol 4). The concentrated solutions containing enzymes in mKBS were brought up to 200 µl with mKBS and eATP substrate was added. eATP degradation was examined in: 1) nominally Mg^2+^-free solution (*mKBS-A*), 2) Ca^2+^-free KBS with 5 mM EGTA (*mKBS-B*), 3) Ca^2+^-free and Mg^2+^-free KBS with 5 mM EGTA (*mKBS-C*), 4) Ca^2+^-free KBS with 5 mM EGTA and 5 mM EDTA (*mKBS-D*), and 5) Ca^2+^-free and Mg^2+^-free KBS with EGTA and EDTA (5 mM each) (*mKBS-E*) (Table 1). Time-courses of enzymatic reactions following addition of eATP to reaction solutions were performed as described in Section 2.4.1 General Protocol of Enzymatic Reactions. The hydrolysis of eATP in mKBS was compared with eATP hydrolysis in regular KBS processed in the same manner as concentrated mKBS. In some experiments, the hydrolysis of eADP was evaluated in mKBS that lacked Mg^2+^ (mKBS-A) or Ca^2+^ (mKBS-B and mKBS-E) and was compared with the eADP hydrolysis in regular KBS.

**TABLE 1:** Modified KBS solutions (mKBS) in which activities of released eATPase(s) were assayed.

	Ca^2+^ (1.8 mM)	Mg^2+^ (1.2 mM)	EGTA (5 mM)	EDTA (5 mM)
KBS	+	+	−	−
mKBS-A	+	−	−	−
mKBS-B	−	+	+	−
mKBS-C	−	−	+	−
mKBS-D	−	+	+	+
mKBS-E	−	−	+	+

### 2.5 Pharmacological Characterization of Nucleotidase Activities

To assess the effects of nucleotidase inhibitors, enzymatic reactions were conducted as described in Section 2.4 Evaluation of Nucleotidase Activities in Suburothelium/Lamina Propria of Detrusor-Free Bladder Preparations either in the presence of vehicle (KBS or DMSO 0.1% in KBS) or of the following nucleotidase inhibitors: ARL67156 (100 µM in KBS; non-specific ENTPDase inhibitor), POM-1 (100 µM in KBS; non-specific ENTPDase inhibitor), ENPP1 Inhibitor C (50 µM in DMSO 0.1%), PSB06126 (10 µM in DMSO 0.1%; ENTPD3 inhibitor), and (-)-p-bromotetramisole oxalate (L-p-BT) (100 µM in DMSO 0.1%; TNAP inhibitor). Bladder preparations were incubated with the inhibitors throughout dissection, equilibration, nondistended and distended conditions and during the time-course reactions.

### 2.6 *Ex vivo* Microdialysis of Bladder Suburothelium

*Ex vivo* bladder preparations with intact detrusor smooth muscle were placed in a dissecting dish with oxygenated KBS and pinned by the serosa to the bottom of the dish. A PE-20 catheter was placed through the urethra into the bladder lumen as described in Section 2.3 *Ex vivo* Detrusor-Free Bladder Preparation. A linear probe for microdialysis (MD) with 2-mm dialysis membrane (CMA 30, Harvard Apparatus, Holliston, MA, United States or OP-X-Y, Amuza Inc., San Diego, CA, United States) was carefully implanted between the bladder mucosa and the detrusor (Figure 4A). The bladder preparation was then moved from the dissecting dish to a water-jacketed chamber filled with oxygenated KBS (pH 7.4 and 37°C). A syringe pump (Kent Scientific, Torrington, CT) was used to fill the bladder with oxygenated KBS through the intraluminal catheter. A second syringe pump was used to perfuse the MD probe with substrate solution. The MD probe was first perfused with KBS at 1 μl/min for 1 h to condition the probe, while the bladder preparation was kept empty. Equilibration was followed by perfusion of the MD probe at 1 μl/min with KBS containing 2 µM eATP. Dialysates were collected for 35 min while the bladder was either empty or was filled with KBS at 15 μl/min to a pre-voiding volume determined at time of dissection. After collecting each sample in ice-cold Eppendorf tube, citric buffer (pH 4.0) was added to the dialysate to preserve the purines in the collected sample. The samples were then processed for detection of eATP decrease and e-product increase by HPLC-FLD technique as described in HPLC analysis of 1,*N* ^*6*^-etheno-nucleotides. The location of the MD probe between detrusor and mucosa was verified at the end of each experiment. E-purines were expressed as percent of total purines detected in each sample.

### 2.7 Preparation of 1,*N* ^*6*^-Etheno-Nucleotides

1,*N* ^*6*^-etheno-ATP (eATP), 1,*N* ^*6*^-etheno-ADP (eADP), 1,*N* ^*6*^-etheno-AMP (eAMP), and 1,*N* ^*6*^-etheno-adenosine (eADO) were prepared according to a modified method of Levitt et al., 1984. Briefly, 180 µl of a citrate phosphate buffer (pH 4.0) containing 62 parts 0.1 M citric acid and 38 parts 0.2 M Na_2_HPO_4_, was added to 150 µl of 200 µM authentic purine (i.e., ATP, ADP, or AMP) in an Eppendorf tube. 2-Chloroacetaldehyde was synthesized according to a modified method of Levitt et al., 1984: equal amounts of 10-fold diluted H_2_SO_4_ in distilled H_2_O and chloroacetaldehyde dimethyl acetal were added to a round bottom boiling flask. The mixture was distilled slowly under a fume hood and the distillate fraction containing approximately 1.0 M 2-chloroacetaldehyde was collected at 79–82°C. The reagent was stored at −20°C when not in use. Twenty µl 2-chloroacetaldehyde was added to the Eppendorf tube containing the mixture of authentic purine and citric buffer in a fume hood and then heated for 40 min at 80°C in a dry bath incubator (Fisher Scientific, United States) to produce 1,*N* ^*6*^-etheno-purine substrates and 1,*N* ^*6*^-etheno-purine standards (Bobalova et al., 2002).

### 2.8 HPLC Analysis of 1,*N* ^*6*^-Etheno-Nucleotides

A reverse phased gradient Agilent 1,200 liquid chromatography system equipped with a fluorescence detector (FLD) (Agilent Technologies, Wilmington, DE, United States) was used to detect 1,*N* ^6^-etheno-purines as described previously (Durnin et al., 2016; Durnin et al., 2019b). Briefly, the stationary phase consisted of a 25-cm by 4.5-mm (5 µm) silica reversed phase ODS-AM (C_18_) column and a matching direct-connect guard column (YMC America, Inc., Devens, MA, United States). Gradient elution with 0.1 M KH_2_PO_4_ (pH 6.0) and increasing methanol (0–35% over 18 min) was employed. Column and autosampler temperatures were maintained at 25 and 4°C, respectively. Flow rate was 1 ml/min, run time was 20 min and post-run time was 5 min 1,*N* ^6^-etheno-derivatized purines were detected by fluorescence at an excitation wavelength of 230 nm and emission wavelength of 420 nm (Bobalova et al., 2002). ChemStation (v. B04-03) software (Agilent Technologies) was used to analyze areas under the peaks. The lower detection sensitivity for 1,*N* ^6^-etheno-derivatized purines was approximately 10 fmol. 1,*N* ^6^-etheno-derivatized purine standards (0.05–5 pmol) were processed with every set of samples.

### 2.9 Lactate Dehydrogenase Cell Toxicity Assay

Lactate dehydrogenase (LDH) activity (a marker of cell membrane integrity damage Radin et al., 2011) was measured in cELS prepared as described in Section 2.4.3 Soluble/Releasable Nucleotidase Activity in Lamina Propria (Protocol 3) from distended detrusor-free or intact bladder preparations and from detrusor-free preparations treated with 1% Triton X-100 for 30 min (to induce cell lysis). LDH activity was determined using a colorimetric Lactate Dehydrogenase Assay Kit (Abcam, United States, Cat# ab102526). Absorbance was measured in 50 µl samples at optical density of 450 nm in a kinetic mode (every 3 min for 1 h) using Promega^™^ GloMax^®^-Multi Detection System (Promega, Madison, WI, United States). LDH activity in samples was compared with a NADH standard and the assay was validated using a positive LDH control provided by the manufacturer. LDH activity was calculated according to the manufacturer instructions, reported in mU/ml and plotted as mean ± SEM.

### 2.10 Automated Capillary Electrophoresis and Immunodetection With Wes Simple Western

For automated capillary electrophoresis and Western blotting by Wes (ProteinSimple, San Jose, CA, United States), mouse brain, urothelium tissue, and cELS were snap-frozen in liquid N_2_, and stored at −80°C until processed (Li et al., 2018; Xie et al., 2018). cELS samples were prepared as described in Section 2.4.3 Soluble/Releasable Nucleotidase Activity in Lamina Propria (Protocol 3). Urothelium and brain samples were homogenized in ice-cold lysis buffer (mM: 50 Tris–HCl pH 8.0, 60 β-glycerophosphate, 100 NaF, 2 EGTA, 25 sodium pyrophosphate, 1 DTT, 0.5% NP-40, 0.2% sodium dodecyl sulfate and protease inhibitors using a Bullet Blender) (one stainless steel bead per tube, speed 6, 5 min) (Bhetwal et al., 2011). The homogenates were then centrifuged at 16,000 × g, for 10 min at 4°C, and the supernatants stored at −80°C. The mouse brain homogenate was used as positive control. Protein concentrations of the supernatants were determined by the Bradford assay using bovine γ-globulin as standard. Protein levels were analyzed according to the Wes User Guide using a Wes Simple Western instrument from ProteinSimple (www.proteinsimple.com). The samples were mixed with the 5X Fluorescent Master Mix (containing 5X sample buffer, 5X fluorescent standard, and 200 mM DTT) and heated at 95°C for 5 min. The boiled samples, protein ladder, blocking buffer, primary antibodies, ProteinSimple horseradish peroxidase-conjugated anti-rabbit or anti-sheep (Invitrogen) secondary antibodies, luminol-peroxide, and wash buffer were loaded into the Wes plate (Wes 12–230 kDa Pre-filled Plates with Split Buffer, ProteinSimple). The plates and capillary cartridges were loaded into the Wes instrument, and protein separation, antibody incubation, and imaging were performed using default parameters. Compass software (ProteinSimple) was used to acquire the data, and to generate the virtual blot image reconstruction and chemiluminescence signal intensity electropherograms. The electropherogram shows the intensity detected along the length of the capillaries, and shows automatically detected peaks, that can be quantified by calculation of the area under the curve (AUC). The lane view is a virtual blot generated by the software from the actual data output, which takes the form of chemiluminescence signals versus apparent MW. Protein levels are expressed as the AUC of the peak chemiluminescence intensity.

#### 2.10.1 Antibodies

The following primary antibodies were used for Wes analysis: from Cell Signaling Technology, rabbit anti-ENTPD1, and (E2X6B); rabbit anti-NT5E, (D7F9A); from ThermoFisher, sheep anti-ENTPD2, and (PA5-47777); rabbit anti-ENTPD3, (PA5-87888); rabbit anti-ENPP1, (PA5-17097); rabbit anti-ENPP3, (PA5-67955); from EpiGentek, rabbit anti-ENTPD8, and (A62482); from Abcam, rabbit anti-NT5C1A, and (ab190214); and, from Novus Biologicals, rabbit anti-TNAP, (SA40-00).

### 2.11 Drugs and Reagents

Adenosine, ATP, ADP, AMP, chloroacetaldehyde dimethyl acetal, dimethyl sulfoxide (DMSO), and Triton X-100 (Sigma-Aldrich, St. Louis, MO, United States), Ethylene glycol-bis(2-aminoethylether)-N,N,N′,N′-tetraacetic acid (EGTA) (ChemCruz Biomedicals, Dallas, TX, United States); N,N′-1,2-Ethanediylbis [N-(carboxymethyl)]glycine (EDTA) (Invitrogen, ThermoFisher Scientific), 6-[(3-aminophenyl)methyl]-N,N,5-trimethyl-[1,2,4]triazolo [1,5-a]pyrimidin-7-amine (ENPP1 Inhibitor C) (Cayman Chemicals, Ann Arbor, MI), (-)-p-bromotetramisole oxalate (L-p-BT) (MedChemExpress, Monmouth Junction, NJ, United States); 6-N,N-Diethyl-

D-β,γ-dibromomethyleneATP trisodium salt (ARL67156), sodium metatungstate (POM-1), and 1-Amino-4-(1-naphthyl)aminoanthraquinone-2-sulfonic acid sodium salt (PSB06126) (Bio-Techne Tocris, Minneapolis, MN)

### 2.12 Data Analysis

Data presented are means ± SEM. In some linear XY graphs, the data points are so similar that the symbols from individual points are superimposed. In some cases, the SEMs lie within the symbol. Scattered plot analysis was performed when appropriate. Means are compared by one-way ANOVA (Wes data) or two-way ANOVA (substrate degradation) for comparison of more than two groups followed by Dunnett’s, Tukey’s or Sidak’s multiple comparisons tests per GraphPadPrism, v. 8.4.2., GraphPad Software, Inc., San Diego, CA. A probability value less than 0.05 was considered statistically significant.

## 3 Results

### 3.1 The Detrusor-Free Bladder Preparation Does Not Display Abnormal Cell Damage of the Anti-Luminal Surface

LDH is a cytoplasmic enzyme present in most cells that is released upon damage of the cytoplasmatic membrane. We tested the LDH activity in cELS collected from detrusor-free preparations and from preparations with intact bladder walls as commonly used in bladder research. We compared the cell damage (measured as LDH activity) of the two types of preparations with cELS collected from detrusor-free bladders that were treated with Triton-X to induce a significant cell damage. The LDH activity was 186.39 ± 41.88 mU/ml in samples from Triton-X-treated preparations and 12.92 ± 2.69 and 50.53 ± 10.9 mU/ml in samples from distended detrusor-free and intact preparations, respectively (*n* = 4 and *P* = 0.9638). The LDH activity was 7.04 ± 0.47% and 30.97 ± 10.95% in cELS of detrusor-free and intact preparations (*p* > 0.05) when compared with the LDH activity in Triton-X treated samples taken as 100%. This data suggests that the detrusor-free bladder preparation does not display greater cell damage than effects that normally occur in commonly-used *ex vivo* bladder dissection.

### 3.2 The degradation of eATP and eADP in the Lamina Propria of filled bladders exceeds the degradation of eATP and eADP in the Lamina Propria of empty bladders

#### 3.2.1 Protocol 1

**FIGURE 3:**
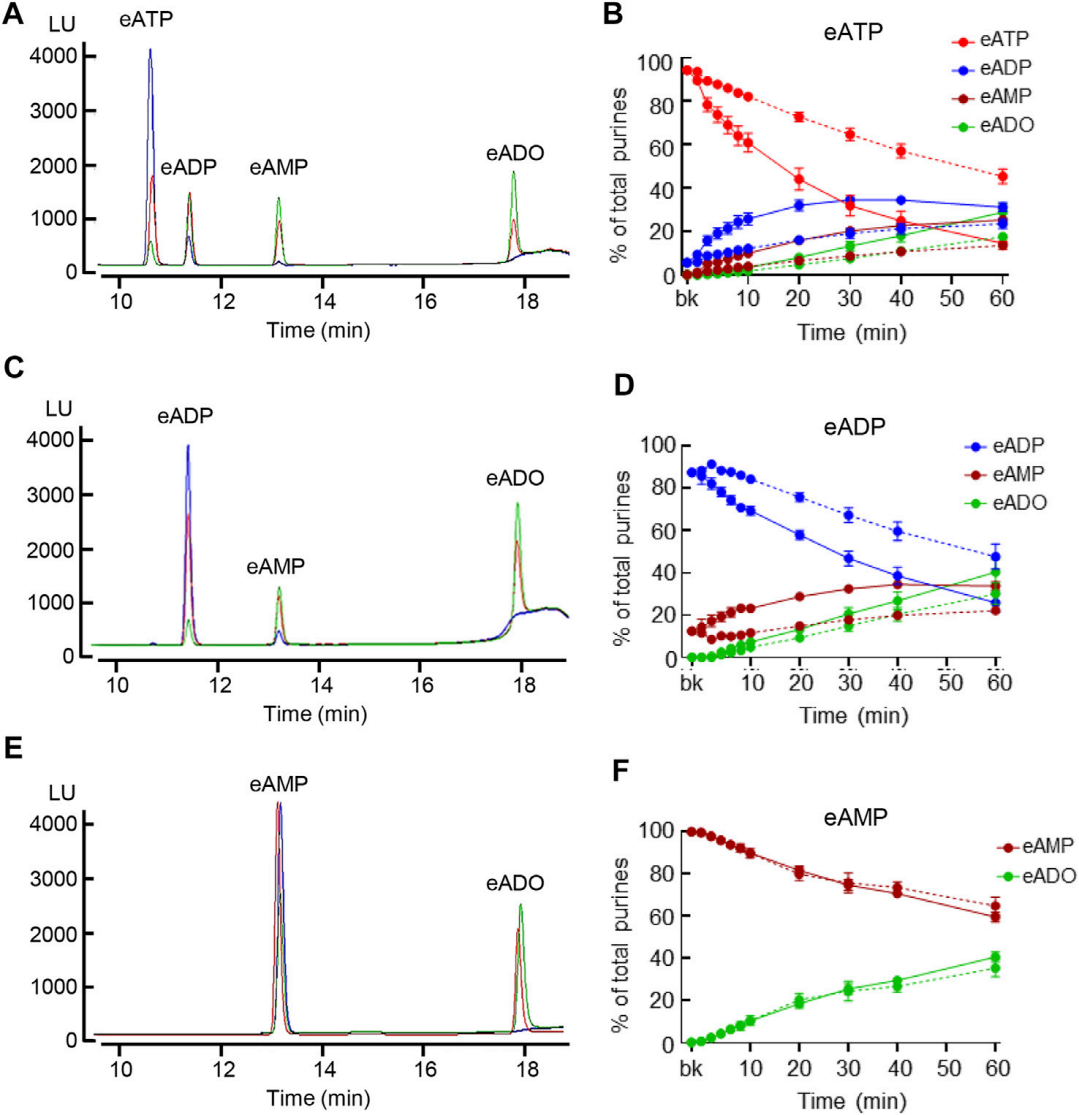
Hydrolysis of eATP, eADP, and eAMP in contact with the LP of nondistended and distended detrusor-free bladders. **(A)** Original chromatograms of eATP in beaker (blue) and at 60 min of contact of eATP with the basolateral/anti-luminal side of nondistended (red) and distended (green) bladder preparations. Note the greater decrease of eATP and greater increase in eADP, eAMP, and eADO in distended than in nondistended preparations. **(B)** Summarized data showing the degradation of eATP in EL samples collected for 1 h after addition of eATP to ELS in the presence of bladder preparation (Protocol 1). eATP, eADP, eAMP, and eADO are presented as percentages of total purines (eATP + eADP + eAMP + eADO) present in ELS samples from nondistended (dotted connecting lines) and distended (solid connecting lines) preparations. **(C)** Original chromatograms of eADP in beaker (blue) and at 60 min of contact of eADP with the anti-luminal side of nondistended (red) and distended (green) bladder preparations. The chromatograms demonstrate greater decrease of eADP and greater increase in eAMP and eADO in distended than in nondistended preparations. **(D)** Summarized data showing the degradation of eADP in EL samples collected at different time points during 1 h after addition of eADP to ELS in the presence of bladder preparation (Protocol 1). eADP, eAMP, and eADO are presented as percentages of total purines (eADP + eAMP + eADO) present in ELS samples from nondistended (dotted connecting lines) and distended (solid connecting lines) preparations. **(E)** Original chromatograms of eAMP in beaker (blue) and at 60 min of contact of eAMP with the anti-luminal side of nondistended (red) and distended (green) bladder preparations. The chromatograms demonstrate similar decrease of eAMP and increase in eADO in distended and nondistended preparations. **(F)** Summarized data showing the degradation of eAMP in EL samples collected during 1 h after addition of eAMP in ELS in the presence of bladder preparation. eAMP and eADO are presented as percentages of total purines (eAMP + eADO) present in ELS samples from nondistended (dotted connecting lines) and distended (solid connecting lines preparations). Statistical significance is described in main text Results.

To determine the degradation of eATP, eADP, and eAMP in contact with the LP, each substrate was added to the bath containing nondistended or distended detrusor-free bladder preparation. As shown in Figure 3, each substrate was progressively reduced and the corresponding products were progressively increased during the contact of substrate and bladder preparations (*n* = 5 in each group). In nondistended preparations, eATP comprised 94.19 ± 1.13% of total purines in beaker (0 min) and reached 45.39 ± 3.33% at 60 min (*p* < 0.0001) of reaction. In distended preparations, a significant decrease of eATP was observed at earlier time points than in nondistended preparations: eATP was reduced from 94.39 ± 0.9% of total purines in beaker to 14.55 ± 2.87% at 60 min (*p* < 0.0001) of reaction, 2wayANOVA with Dunnett’s multiple comparisons test. The eATP decrease in nondistended preparations reached statistical significance at 8 min, whereas in distended preparations the decrease of eATP was statistically significant at 2 min of reaction (*p* < 0.05). The eATP decrease was significantly greater in distended than in nondistended preparations for the entire time of reaction starting at 4 min (*P* = 0.0170), 2way ANOVA Sidak’s multiple comparisons test. The increase of the eATP products eADP, eAMP, and eADO was also greater in distended than in nondistended preparations. Thus, at 60 min of reaction, eADP was 23.54 ± 2.29% and 31.23 ± 2.03% (*P* = 0.0637); eAMP was 13.56 ± 0.98% and 25.31 ± 1.27% (*p* < 0.0001), and eADO was 17.5 ± 1.28% and 28.9 ± 4.09% (*p* < 0.0001) in nondistended and distended preparations, respectively; 2way ANOVA Sidak’s multiple comparisons test.

Similar results were obtained with eADP as substrate (Figures 3C,D). eADP was decreased from 87.57 ± 1.14% in beaker to 47.7 ± 6.02% at 60 min (*p* < 0.0001) of reaction in nondistended preparations and from 87.56 ± 1.14% in beaker to 25.86 ± 4.4% at 60 min (*p* < 0.0001) of reaction in distended preparations, 2way ANOVA, Tukey multiple comparisons test. The eADP decrease was statistically significant at 10 min and at 30 min of reaction in distended and non-distended preparations, respectively (*p* < 0.05 vs. 0 min). The eADP decrease was greater in distended than in nondistended preparations during the enzymatic reaction starting at 6 min (*P* = 0.0153), *n* = 4 in each group, 2way ANOVA with Sidak’s multiple comparisons test. The increase of the eADP products eAMP and eADO was also greater in distended than in nondistended preparations. Thus, at 60 min of reaction, eAMP was 22.18 ± 1.29% and 33.79 ± 2.05% in nondistended and distended preparations, respectively (*p* < 0.0001) whereas eADO was 30.12 ± 5.19% and 40.35 ± 5.93% in nondistended and distended bladders, respectively (*P* = 0.0277).

The eAMP substrate (Figures 3E,F) was decreased from 99.78 ± 0.07% in beaker to 64.71 ± 4.17% and 59.45 ± 2.32% at 60 min of reaction in nondistended and distended preparations respectively, *n* = 4 in each group, *p* < 0.0001 vs. 0 min, 2way ANOVA Tukey’s multiple comparisons test. No significant differences were observed in eAMP decrease or eADO increase between nondistended and distended preparations at all time points of reaction (*p* > 0.05), 2way ANOVA Sidak’s multiple comparisons test.

### 3.3 Application of eATP in the Suburothelium *via* Microdialysis Results in eATP Hydrolysis

**FIGURE 4:**
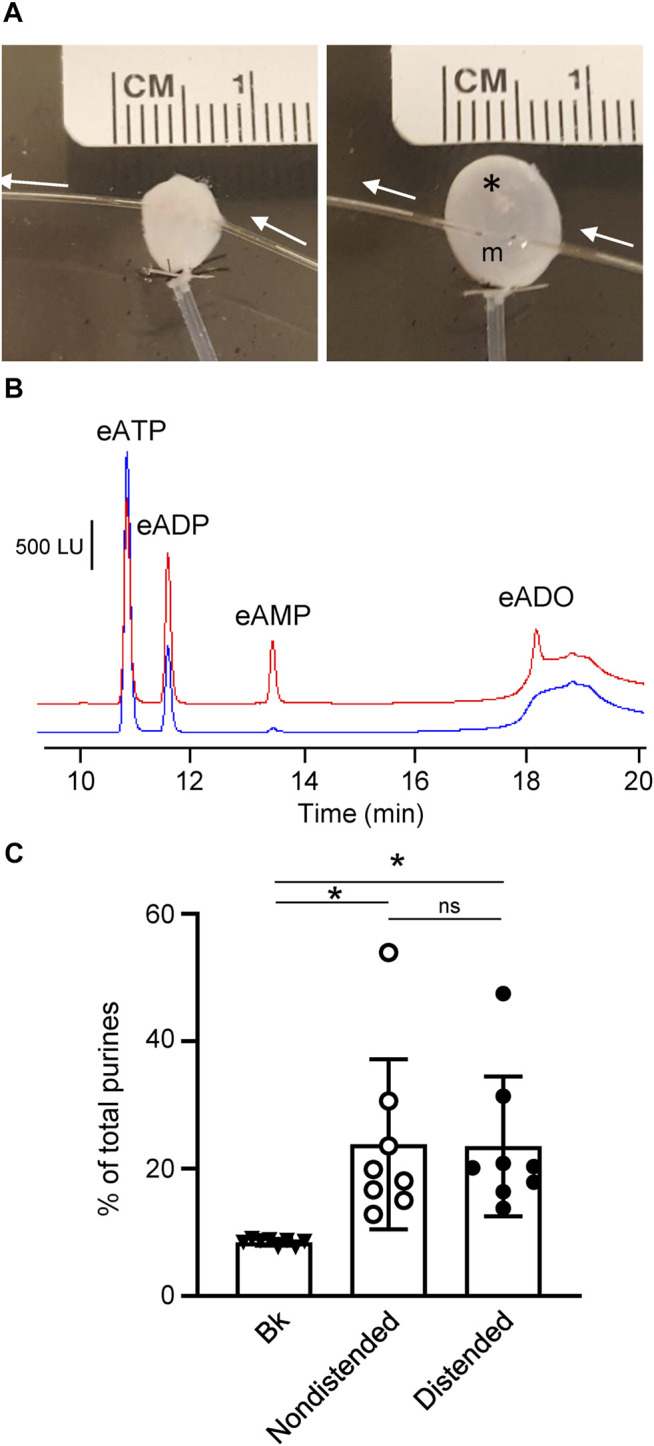
Degradation of eATP in the suburothelium evaluated by microdialysis of the bladder wall. **(A)** Microdialysis probe implanted between suburothelium/LP and detrusor muscle of nondistended (left panel) and distended (right panel) mouse urinary bladder. m, microdialysis membrane outlined with black dots; white arrows show the direction of microdialysis perfusion. *, air bubbles inserted in bladder to show transparency of the wall. **(B)** Original chromatograms of eATP substrate in absence of tissue (beaker and blue) and in dialysate from nondistended bladder (red). Addition of eATP in the solution perfusing the microdialysis probe resulted in an eADP increase and the appearance of eAMP and eADO. **(C)** Summarized data showing formation of product (eADP + eAMP + eADO) from eATP after microdialysis of nondistended and distended bladder preparations with eATP. Data are presented as percentages of total purines (eATP + eADP + eAMP + eADO) present in dialysate. *P < 0.05, ns, non-significant difference.

To determine whether eATP degradation occurs in the LP in the presence of detrusor, we applied the eATP substrate (2 µM) in the suburothelium using a MD probe inserted between urothelium and detrusor and examined whether an eATP decrease and product increase can be detected in dialysate (Figure 4 **)**. eATP was decreased from 91.56 ± 0.18% of total purines in beaker to 76.83 ± 4.84% (*P* = 0.0361) in dialysate from nondistended preparations (*n* = 8). eADP was 8.085 ± 0.18% and 12.87 ± 1.92% in beaker and dialysate, respectively (*P* = 0.0839) whereas eAMP was increased from 0.36 ± 0.002% in beaker to 6.88 ± 2.34% in dialysate (*P* = 0.0478). eADO was also significantly increased from 0% in beaker to 4.057 ± 0.596% of total purines in dialysate from nondistended preparations (*p* < 0.0005); one-way ANOVA with Dunnett’s multiple comparisons test. We also measured the degradation of eATP during microdialysis of a bladder that was filled with KBS at 15 μl/min. Similar to nondistended preparations, eATP degradation was observed in dialysates from distended preparations: thus, eATP was reduced to 77.58 ± 4.15% (*P* = 0.0237) and eADP, eAMP, and eADO were increased to 13.13 ± 1.65% (*P* = 0.0403), 6.66 ± 1.94% (0.0253), and 3.71 ± 0.41% (<0.0001), respectively, of total purines in dialysates. The total product formation in dialysate was significantly greater than the total eATP product in beaker. There was no significant difference in product formation in dialysates from nondistended and distended preparations, *p* > 0.05 (Figure 4C), One-way ANOVA with Tuckey’s multiple comparisons test. This data confirmed the key observation in detrusor-free bladder preparations signifying that ATP is hydrolyzed in the suburothelium to ADP, AMP, and ADO.

### 3.4 Enzymes That Hydrolyze eATP and eADP are Released in the Lamina Propria in a Stretch-Dependent Manner

#### 3.4.1 Protocol 2

**FIGURE 5:**
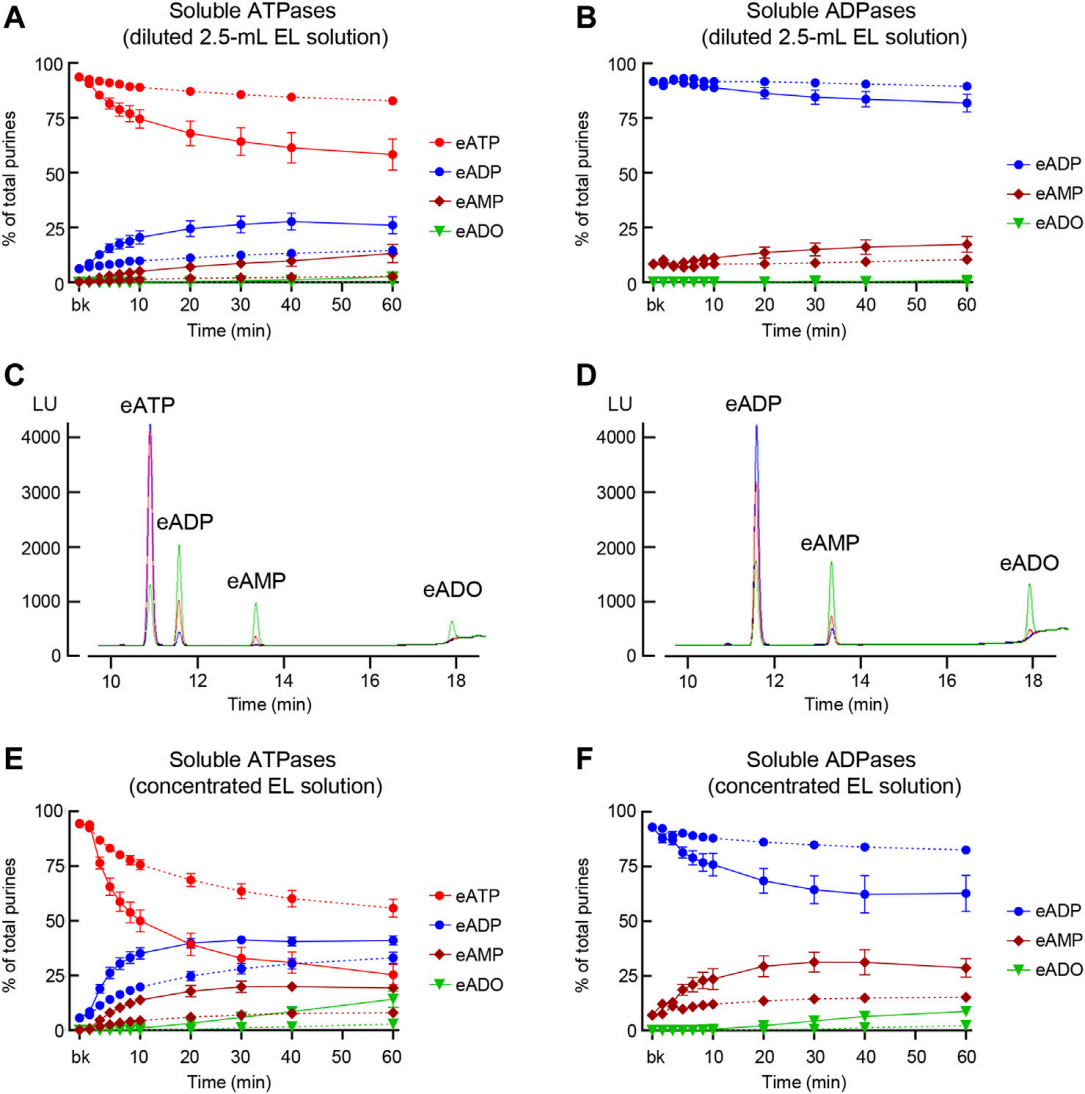
Degradation of eATP and eADP by soluble enzymes released in the LP at rest and during bladder filling. **(A)** Summarized data showing the degradation of eATP in diluted (i.e., 2.5-ml) EL samples collected at different time points during 1 h after addition of eATP in the absence of bladder preparation (Protocol 2). eATP, eADP, eAMP, and eADO are presented as percentages of total purines (eATP + eADP + eAMP + eADO) present in EL solution samples from nondistended (dotted connecting lines) and distended (solid connecting lines) preparations. **(B)** Summarized data showing the degradation of eADP in diluted (i.e., 2.5-ml) EL samples collected at different time points during 1 h after addition of eADP in the absence of bladder preparation (Protocol 2). eADP, eAMP, and eADO are presented as percentages of total purines (eADP + eAMP + eADO) present in EL solution samples from nondistended (dotted connecting lines) and distended (solid connecting lines) preparations. **(C)** Original chromatograms of eATP in beaker (blue) and at 60 min of addition of eATP to concentrated EL samples (Protocol 3) collected from nondistended (red) and distended (green) bladder preparations. The chromatograms demonstrate greater decrease of eATP and increase in eADP, eAMP, and eADO in EL solutions from distended than from nondistended preparations. **(D)** Original chromatograms of eADP in beaker (blue) and at 60 min of addition of eADP to concentrated EL samples (Protocol 3) collected from nondistended (red) and distended (green) bladder preparations. The chromatograms demonstrate greater decrease of eADP and increase in eAMP and eADO in EL solutions from distended than from nondistended preparations. **(E)** Summarized data showing the degradation of eATP in EL samples collected at different time points during 1 h after addition of eATP in concentrated EL samples collected from nondistended (dotted connecting lines) and distended (solid connecting lines) (Protocol 3). eATP, eADP, eAMP, and eADO are presented as percentages of total purines (eATP + eADP + eAMP + eADO) present in the EL solution samples. **(F)** Summarized data showing the degradation of eADP in EL samples collected at different time points during 1 h after addition of eADP in concentrated EL samples collected from nondistended (dotted connecting lines) and distended (solid connecting lines) preparations (Protocol 3). eADP, eAMP, and eADO are presented as percentages of total purines (eADP + eAMP + eADO) present in the EL solution samples. Statistical significance is described in main text Results.

To determine whether soluble enzymes contribute to distention-dependent hydrolysis of eATP and eADP in the LP, the substrate was added to large-volume EL solutions that were transferred from chambers containing nondistended or distended bladders to empty chambers. The final volume of ELS plus substrate was 2.5 ml. Substrate decrease and product increase was measured for 1 h following addition of substrate. As shown in Figure 5A, eATP was significantly decreased in solutions collected from distended preparations, but not in solutions collected from nondistended preparations (*n* = 12 in each group). Thus, at 60 min of reaction, eATP was 93.63 ± 0.61% in beaker, 82.81 ± 1.93% in solutions from nondistended preparations (*P* = 0.4125) and 58.29 ± 7.09% in solutions from distended preparations (*p* < 0.0001), 2way ANOVA with Tukey’s multiple comparisons test. The decrease of eATP was greater in solutions from distended than from nondistended preparations at 10–60 min (*p* < 0.05), 2way ANOVA with Sidak’s multiple comparisons test. Likewise, the formation of eADP at 6–60 min, of eAMP at 30–60 min, and of eADO at 60 min was significantly greater (*p* < 0.05) in solutions collected from distended than from nondistended detrusor-free preparations.

When eADP was used as substrate in 2.5 ml ELS (Figure 5B), a significant decrease of eADP from beaker was observed at 40 min (*P* = 0.0406) and 60 min (*P* = 0.0044) of enzymatic reaction in ELS from distended but not from nondistended preparations (*n* = 4, 2way ANOVA with Tukey’s multiple comparisons test). The difference of eADP decrease in ELS from nondistended and distended bladders was significant at 40 min (*P* = 0.0486) and 60 min (*P* = 0.0197) of reaction, 2way ANOVA with Sidak’s multiple comparisons test.

#### 3.4.2 Protocol 3

Prominent eATP or eADP substrate decrease and product increase was revealed in 12.5-fold concentrated ELS (cELS) from both nondistended and distended preparations (Figures 5C–F). In cELS from nondistended bladders (Figure 5E), a significant decrease of eATP was observed in 8–60 min (*p* < 0.05) as compared with eATP in beaker. In cELS from distended preparations, however, a significant decrease of eATP (*p* < 0.05) was observed at all time points except at 10 s (*n* = 15, 2way ANOVA Tuckey multiple comparisons tests). The decrease of eATP was significantly greater in cELS from distended than from nondistended preparations at 4–60 min of reaction (*p* < 0.05), 2way ANOVA Sidak’s multiple comparisons test. The formation of eADP and eAMP from eATP was significantly greater in cELS from distended than from nondistended preparations at 4–60 min and 6–60 min, respectively (*p* < 0.05 and 2way ANOVA Sidak’s multiple comparisons test). The increase in eADO was significant at 30–60 min (*p* < 0.05) of reaction in cELS from distended preparations, but it did not reach significance at all time points in cELS from nondistended preparations, 2way ANOVA Tukey’s multiple comparisons test (Figure 5E).

eADP was not significantly degraded in cELS from nondistended preparations (Figures 5D,F). In cELS from distended preparations, however, a significant decrease of eADP was observed from 92.88 ± 0.62% in beaker to 62.7 ± 8.23% at 60 min of reaction (*p* < 0.0001), *n* = 4, 2way ANOVA, and Sidak’s multiple comparisons tests. The eADP decrease was statistically significant at 20–60 min. Significant increase of eAMP and eADO was observed at 6–60 min and 30–60 min, respectively (*p* < 0.0001 at 60 min for both products), *n* = 4, 2way ANOVA Sidak’s multiple comparisons tests (Figure 5F).

### 3.5 Activities of Released Nucleotidases Depend Differentially on Extracellular Ca^2+^ and Mg^2+^

#### 3.5.1 Protocol 4

**FIGURE 6:**
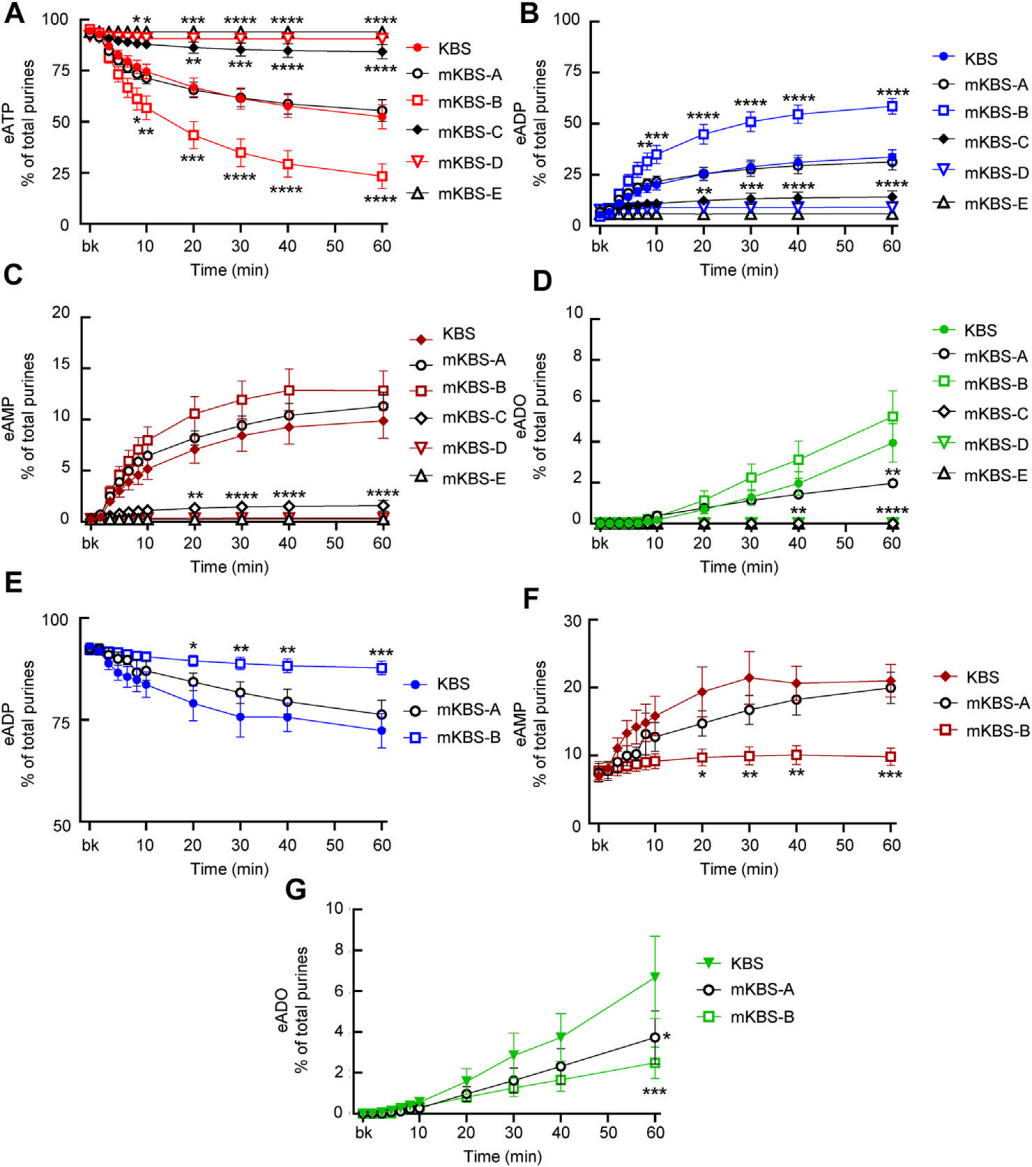
Effects of extracellular Ca^2+^ and Mg^2+^ on activities of released ATPases and ADPases. **(A–D)** eATP hydrolysis by soluble enzymes in controls (KBS) and mKBS: panel **(A)** shows the decrease of eATP and panels **(B–D)** show the increase in eADP, eAMP, and eADO formed from eATP, respectively. **(E–G)** eADP hydrolysis by soluble enzymes in control (KBS) and mKBS: panel **(E)** shows eADP decrease and panels **(F,G)** show the increase in eAMP and eADO formed from eADP. KBS contains normal Ca^2+^ (1.8 mM) and Mg^2+^ (1.2 mM). mKBS-A contains normal Ca^2+^ (1.8 mM) and no Mg^2+^ (0 mM); mKBS-B contains 0 mM Ca^2+^, normal Mg^2+^ (1.2 mM), and EGTA (5 mM); mKBS-C contains 0 mM Ca^2+^, 0 mM Mg^2+^, and EGTA (5 mM); mKBS-D contains 0 mM Ca^2+^, normal Mg^2+^ (1.2 mM), EGTA (5 mM), and EDTA (5 mM); mKBS-E contains 0 mM Ca^2+^, 0 mM Mg^2+^, EGTA (5 mM), and EDTA (5 mM). Asterisks denote significant difference from controls (KBS). *p < 0.05, **p < 0.01, ***p < 0.001, ****p < 0.0001.

To determine how activities of enzymes released in the LP during bladder distention depend on Ca^2+^ and Mg^2+^, we examined the eATP and eADP hydrolysis in mKBS that contained soluble enzymes but lacked Ca^2+^ or Mg^2+^, in the presence of EGTA or EGTA + EDTA (Table 1). Replacement of regular KBS with mKBS required additional centrifugation of ELS prior to performing the enzymatic reactions. The control ELS (KBS) subjected to additional centrifugations retained nucleotidase activities as demonstrated by the substrate decrease and product increase after addition of eATP or eADP (Figures 6A,B).

In control ELS (KBS) from distended preparations (Figure 6A), the substrate eATP was decreased from 94.4 ± 0.24% in beaker to 52.44 ± 5.97% at 60 min (*p* < 0.0001) of reaction (*n* = 9 and 2way ANOVA with Tukey’s, multiple comparisons test). The decrease of eATP remained unchecked in mKBS-A that contained normal Ca^2+^, but lacked Mg^2+^(*n* = 3) (Figure 6A). However, in mKBS-B that contained normal Mg^2+^ but lacked Ca^2+^ (*n* = 5), the decrease of eATP was significantly enhanced at 8–60 min of reaction when compared with KBS controls (Figure 6A). Complete absence of Ca^2+^ in this solution was ensured by addition of 5 mM EGTA that specifically chelates Ca^2+^ (see Discussion). Removal of Mg^2+^ and Ca^2+^ in the presence of EGTA (mKBS-C and *n* = 4) almost completely inhibited the eATP decrease (Figure 6A). eATP hydrolysis was abolished in mKBS to which the non-specific chelator EDTA (5 mM) was added (e.g., mKBS-D and mKBS-E, each *n* = 3), Figure 6A.

The increase of eADP formed from eATP in mKBS followed similar patterns: the eADP increase was not affected by removal of Mg^2+^ alone, was enhanced in the absence of Ca^2+^ plus EGTA, and was abolished in the absence of both Ca^2+^ and Mg^2+^ and addition of EDTA (Figure 6B). The increase of eAMP formed from eATP (Figure 6C) was inhibited in the absence of both Ca^2+^ and Mg^2+^ in the presence of EDTA and/or EGTA, but the eAMP increase was not affected by the absence of either Ca^2+^ or Mg^2+^ alone. eADO formation (Figure 6D) reached statistically significant values at 40 and 60 min of reaction. The eADO increase was reduced at 60 min in the absence of Mg^2+^, but was not changed in Ca^2+^-free solution with EGTA (mKBS-B). As with the other e-products, the formation of eADO from eATP was abolished when both Ca^2+^ and Mg^2+^ were removed or EDTA was added (i.e., mKBS-C, mKBS-D, and mKBS-E) (Figure 6D).

When eADP was used as substrate, it did not decrease significantly in EL solutions collected from nondistended preparations (data not shown). However, in cELS from distended preparations (KBS controls, *n* = 5, Figure 6E), eADP decreased from 92.99 ± 0.78% in beaker to 72.34 ± 4.23% at 60 min (*p* < 0.0001) of reaction. The decrease of eADP was accompanied with an increase of eAMP from 7.01 ± 0.78% in beaker to 20.99 ± 2.39% at 60 min (*p* < 0.0001) of reaction (Figure 6F). The formation of eADO from eADP was significantly increased from 0 ± 0% in beaker to 6.67 ± 2.02% at 60 min (*p* < 0.0001) (Figure 6G). Similar to the eATP substrate, removal of Mg^2+^ alone (mKBS-A) did not affect significantly the degradation of eADP. In contrast to eATP, however, removal of Ca^2+^ and addition of EGTA (i.e., mKBS-B) significantly inhibited the decrease of eADP substrate (Figure 6E). The formation of eAMP from eADP appeared to be reduced in nominally Mg^2+^-free solution (mKBS-A); however, this effect did not reach statistical significance for the duration of 1 h. No formation of eAMP from eADP was observed in Ca^2+^-free solutions containing EGTA (Figure 6F). The formation of eADO from eADP was decreased at 60 min of reaction in mKBS-A lacking Mg^2+^ and in mKBS-B that lacked Ca^2+^ and contained EGTA (Figure 6G).

### 3.6 Inhibitors of Membrane-Bound Nucleotidases Impede the Hydrolysis of eATP and eADP by Soluble Enzymes Released in the Lamina Propria

#### 3.6.1 Effects of ARL67156

**FIGURE 7:**
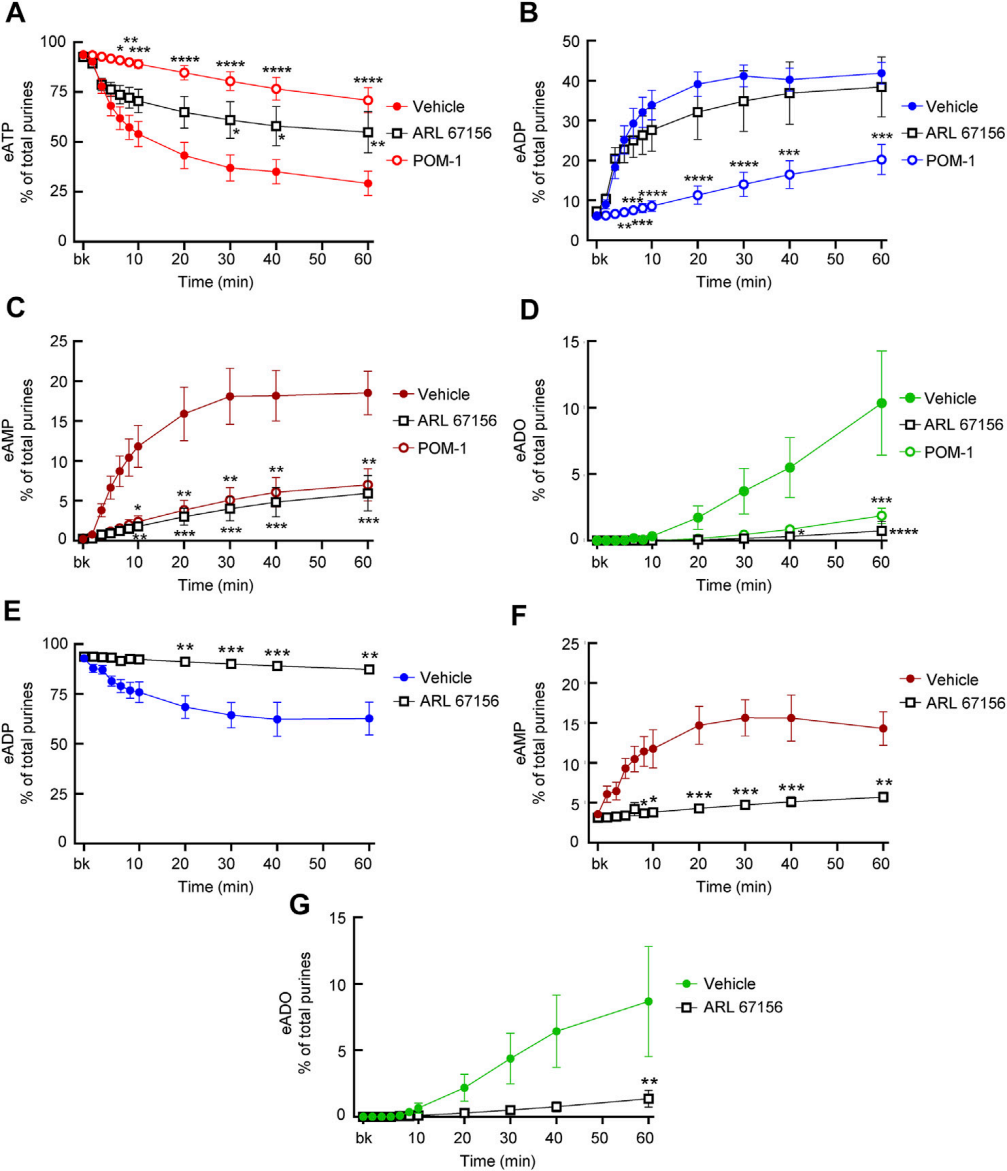
Effects of ARL67156 and POM-1 on hydrolysis of eATP by soluble enzymes in LP. **(A–D)** eATP in the presence of vehicle (KBS) or ARL67156 (100 µM) or POM-1 (100 µM). **(E–G)** eADP hydrolysis by soluble enzymes in the presence of vehicle (KBS) or ARL67156 (100 µM). Asterisks denote significant difference from vehicle controls. *p < 0.05, **p < 0.01, ***p < 0.001, ****p < 0.0001.

ARL67156 is an ATP analog that was introduced as “an ATPase inhibitor” in the 1990s (Crack et al., 1995). As shown in Figure 7A, the degree of eATP decrease in cELS from distended preparations was reduced by ARL67156 (100 µM). The inhibition by ARL67156 was statistically significant at 30–60 min of reaction. At 60 min, eATP was 29.24 ± 6.107% of total purines in controls (*n* = 9) and 46.87 ± 11.35% in the presence of ARL67156 (*n* = 5), *P* = 0.0076. eADP increase was not affected by ARL67156 at all time points of reaction (Figure 7B). However, the increase of eAMP was significantly inhibited in the presence of ARL67156 at 10–60 min of enzymatic reaction (Figure 7C) and the increase in eADO was almost abolished (Figure 7D). When eADP was used as substrate, ARL67156 almost abolished the decrease of eADP and the increase of eAMP and eADO (Figures 7E–G).

#### 3.6.2 Effects of POM-1

POM-1 is a polyoxometalate that inhibits ENTPDases (Muller et al., 2006). As shown in Figure 7, the hydrolysis of eATP was significantly inhibited by POM-1 (100 µM) since the decrease of eATP at 6–60 min of reaction (Figure 7A) and the formation of eADP, eAMP, and eADO (Figures 7B–D) were diminished in the presence of POM-1. POM-1 appeared to have a greater effect on the decrease of eATP than ARL67156 (Figure 7A); however, the differences between the effects of the two drugs did not reach statistical significance. The increase of eADP was diminished in the presence of POM-1 but not in the presence of ARL-67156 (Figure 7B) whereas the formation of eAMP and eADO was decreased by both POM-1 and ARL67156 (Figures 7C,D). These data suggest that POM-1 and ARL67156 differ in inhibiting the hydrolysis of eATP.

#### 3.6.3 Effects of PSB06126

**FIGURE 8:**
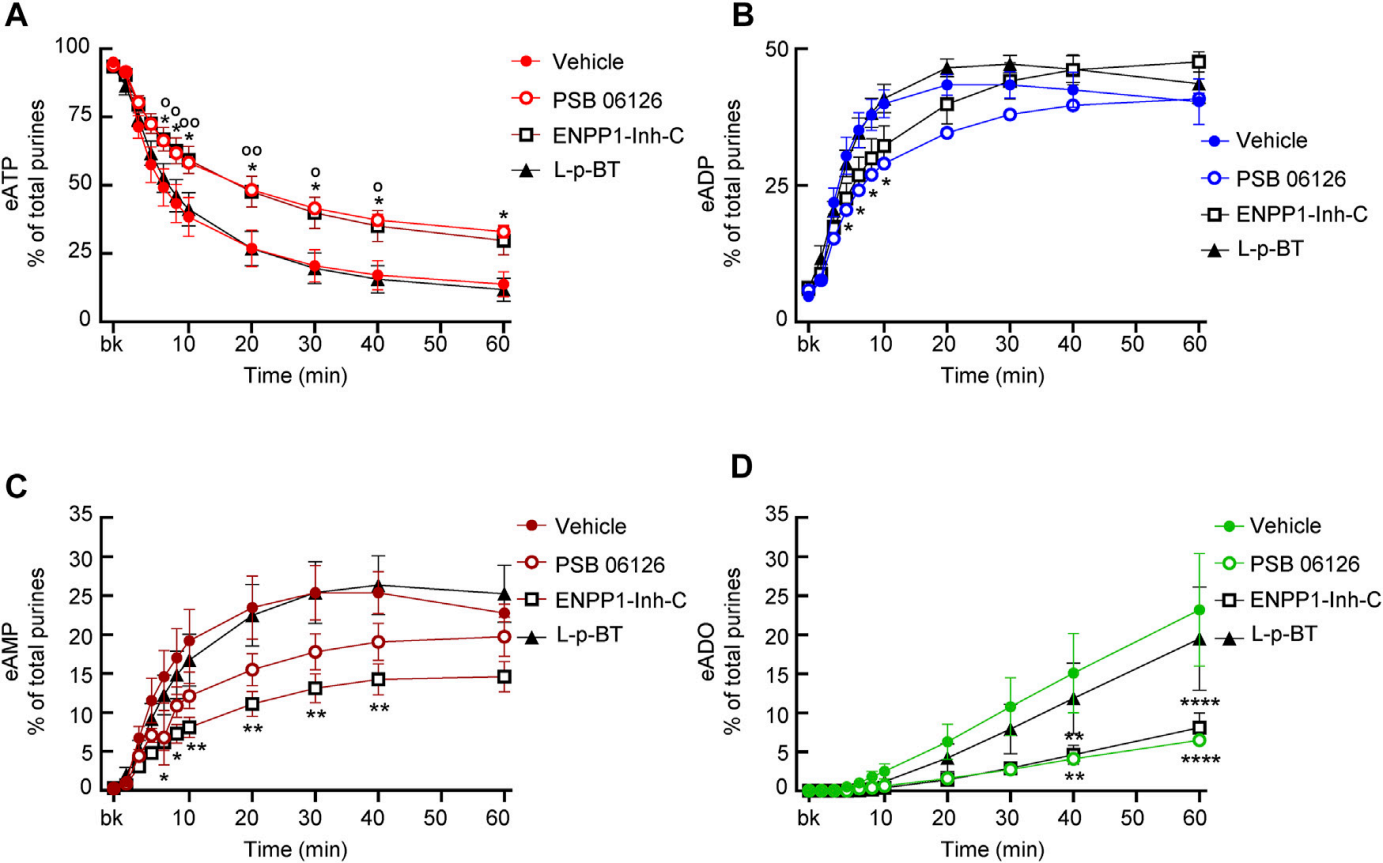
Effects of PSB06126, ENPP1-Inh-C and L-p-bromotetramisole (L-p-BT) on hydrolysis of eATP by soluble enzymes in LP. **(A–D)** eATP hydrolysis by soluble enzymes in the presence of vehicle (0.1% DMSO) or PSB06126 (10 µM), ENPP1-Inh-C (50 µM) or L-p-bromotetramisole (L-p-BT) (100 µM). Asterisks denote significant difference of eATP decrease in the presence of ENPP1-Inh-C from vehicle controls. *p < 0.05, **p < 0.01, ****p < 0.0001. Open circles denote significant difference of eATP decrease in the presence of PSB06126 and vehicle control (panel **A**). ^o^P<0.05, ^oo^P<0.01.

The ENTPD3 inhibitor PSB06126 (10 µM), significantly diminished the decrease of eATP from 6 to 60 min of reaction (Figure 8A), suggesting that a soluble form of ENTPD3 might be released in the LP. The increase of eADP was diminished in the earlier time points (e.g., 4–10 min) but not in the later time points of reaction (Figure 8B). The presence of PSB06126 resulted in less production of eAMP and eADO (Figures 8C,D).

#### 3.6.4 Effects of ENPP1-Inhibitor-C

ENPP1 Inhibitor C diminished the decrease of eATP, did not affect the increase of eADP, and significantly reduced the formation of eAMP and eADO (Figure 8), suggesting that it likely inhibited the direct hydrolysis of eATP to eAMP and subsequently to eADO.

#### 3.6.5 Effects of L-p-BT

The TNAP inhibitor L-p-BT had no significant effect on the eATP decrease or the eADP, eAMP, and eADO increase (Figure 8), suggesting that alkaline phosphatases that are sensitive to this inhibitor do not play a significant role in the degradation of eATP by soluble enzymes in the bladder LP.

### 3.7 Soluble Enzymes in the Lamina Propria are Similar to Known Membrane-Bound Nucleotidases

**FIGURE 9:**
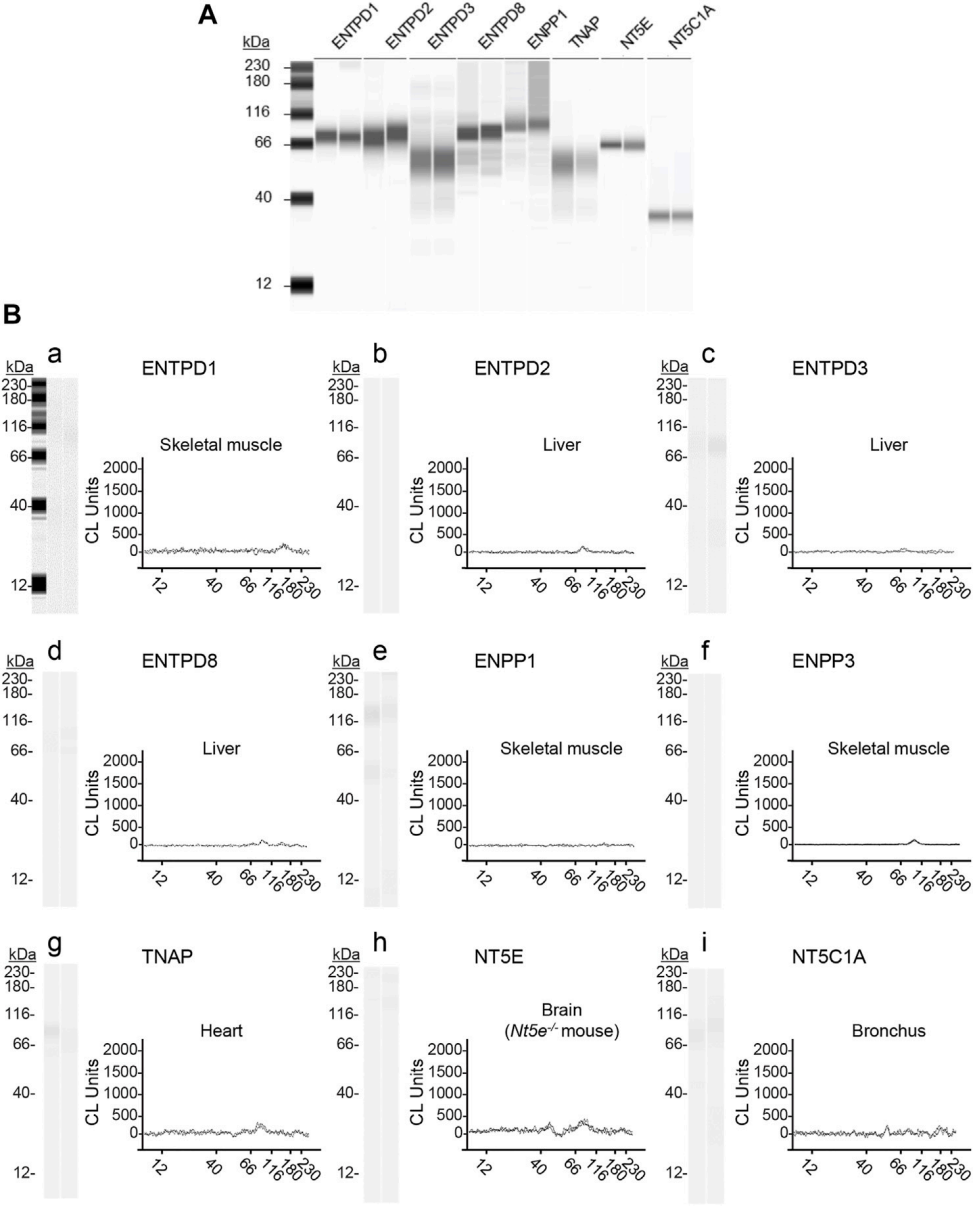
Validation of anti-nucleotidase antibodies used for enzyme identification. ProteinSimple Wes was used to specifically detect the indicated nucleotidases in **(A)** mouse brain homogenates (positive controls) and in **(B)** mouse tissue homogenates in which the indicated nucleotidase has not been detected (negative controls). Panel **(Ba–Bi)** show representative blot images (left) and immunoelectropherograms (right) of negative control tissue homogenates. Each antibody was tested in duplicate. The antibodies, dilutions, corresponding amounts of homogenate per well, and expected molecular weight (from vendor) used were: rabbit anti-ENTPD1 (1:200, 15 μg, and 80 kDa), sheep anti-ENTPD2 (1:500, 7.5 µg, and 80 kDa); rabbit anti-ENTPD3 (1:500, 3.0 µg, and 80 kDa); rabbit anti-ENTPD8 (1:200, 15 μg, and 60 kDa); rabbit anti-ENPP1 (1:500, 15 μg, and 100 kDa); rabbit anti-ENPP3 (1:500, 15 μg, and 100 kDa); rabbit anti-NT5E (1:500, 15 μg, 7and 0 kDa); rabbit anti-TNAP (1:200, 30 μg, and 60 kDa; rabbit anti-NT5C1A (1:100, 30 μg, and 40 kDa)).

**FIGURE 10:**
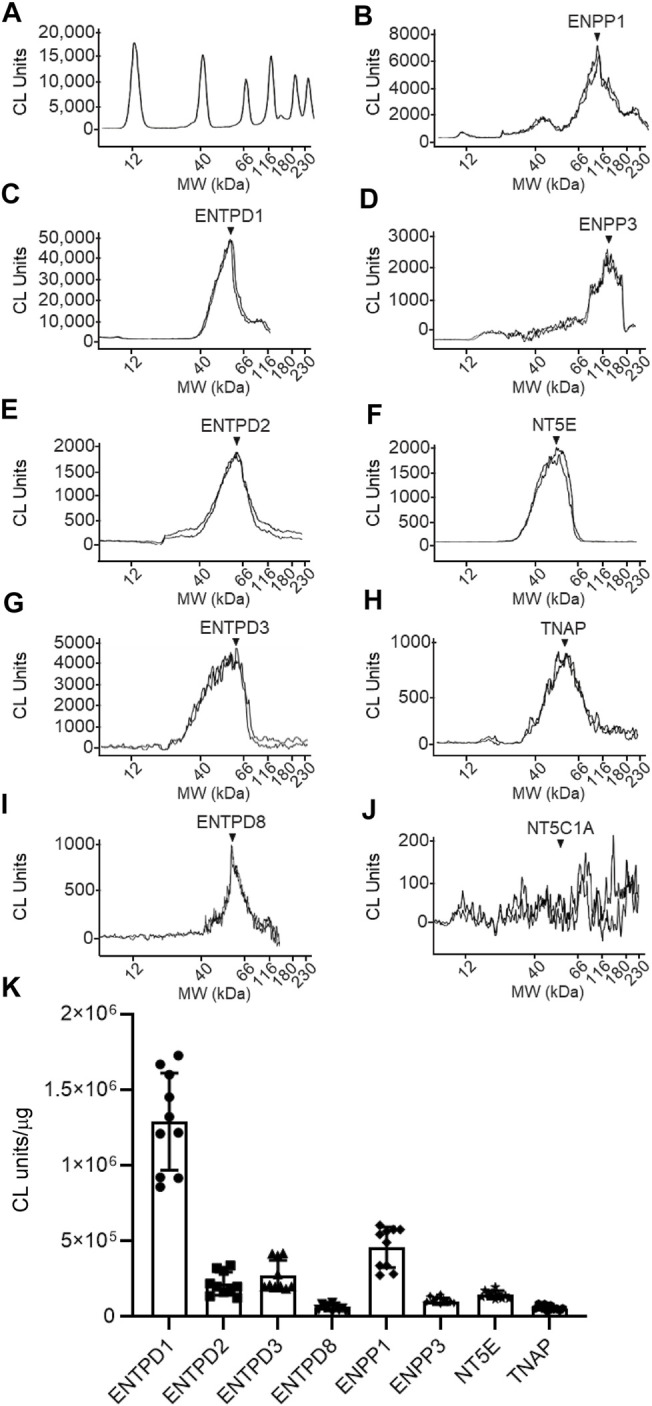
Protein expression levels in urothelium homogenates prepared from detrusor-free bladder preparations. Representative immunoelectropherograms (duplicates) of nucleotidases detected in urothelium using ProteinSimple Wes **(A–J)**. Each antibody was diluted 100-fold and each well contained 6 µg of urothelium homogenate sample. The antibodies used are described in Figure 9 and in main text Antibodies. **(K)** Scatter plots of AUC of chemiluminescence (CL) signals normalized per µg loaded urothelium sample. Each symbol represents a single loading from 5 urothelium samples, loaded in triplicates. Statistical significance is described in main text Results.

**FIGURE 11:**
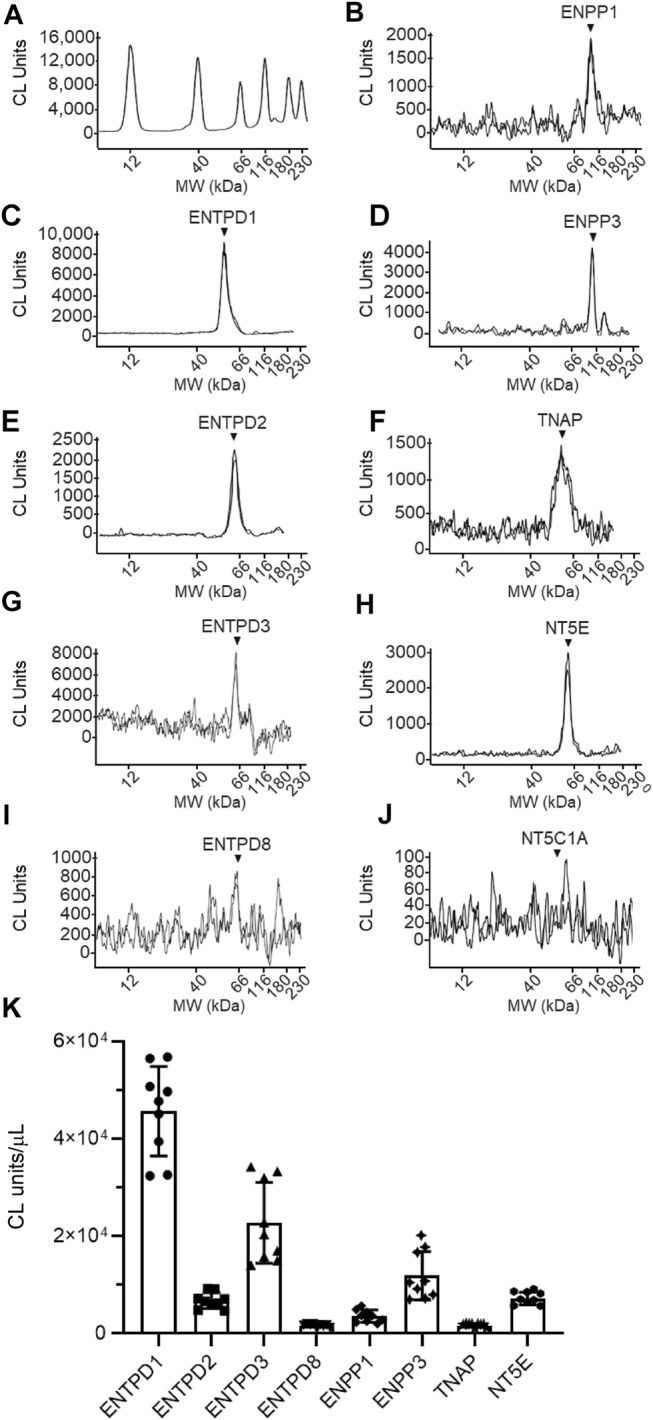
Protein expression levels in cELS collected from distended detrusor-free bladder preparations. Representative immunoelectropherograms (duplicates) of nucleotidases detected in cELS using ProteinSimple Wes **(A–J)**. Each antibody was diluted 100-fold and each well contained 3 µl of cELS sample. The antibodies used are described in Figure 9 and in main text Antibodies. **(K)** Scatter plots of AUC of chemiluminescence (CL) signals normalized per µL loaded cELS sample. Each symbol represents a single loading from 3 cELS samples, loaded in triplicates. Statistical significance is described in main text Results.

Urothelium tissues and cELS collected from distended preparations were investigated by Wes analysis for expression of known membrane-bound nucleotidases that use ATP or ADP as substrates (Figures 10, 11). The antibodies used for identification of ENTPD1, ENTPD2, ENTPD3, ENTPD8, ENPP1, ENPP3, NT5E, TNAP, and NT5C1A were validated in mouse brain homogenates used as positive controls (Figure 9A). In addition, each antibody was tested in tissue homogenates in which the indicated nucleotidase has not been detected per The Mouse Gene Expression Database (http://www.informatics.jax.org/expression.shtml) and The Human Protein Atlas (https://www.proteinatlas.org). Thus, tissue homogenates from skeletal muscle, liver, heart, and bronchus as well as from brain of a *Nt5e* ^*−/−*^ mouse were used as negative controls for antibody validation. No signals were detected in the negative control tissue homogenates (Figure 9B) using the same antibody dilutions and amounts of negative control tissue homogenates that were used for the corresponding mouse brain homogenate positive controls (Figure 9A). These experiments validated that the signals from the urothelium homogenates (Figure 10) and cEL samples (Figure 11) are due to binding of the antibodies to their respective target antigens, and not from non-specific antibody interactions, or false-positives.

The following enzymes were detected in both urothelium tissue homogenates (Figure 10) and cEL samples from distended preparations (Figure 11): ENTPD1, ENTPD2, ENTPD3, ENTPD8, ENPP1, ENPP3, NT5E, and TNAP. Note that NT5C1A was not resolved in urothelium, and was not detected in the EL samples, indicating that NT5C1A does not contribute to the pool of released enzymes in the LP. ENTPD1 was the main nucleotidase expressed in urothelium and in cELS (*p* < 0.0001 from all other nucleotidases in each sample kind) whereas ENTPD8 and TNAP were barely detected in both types of samples. Importantly, the relative expression of nucleotidases differed in urothelium and cELS. In the urothelium, ENPP1 was the second highly expressed protein and was significantly higher than ENTPD2 (*P* = 0.0003), ENTPD3 (*p* < 0.0001), ENPP3 (*p* < 0.0001), NT5E (*p* < 0.0001), ENTPD8 (*p* < 0.0001), and TNAP (*p* < 0.0001), 2way ANOVA with Tuckey multiple comparisons test (Figure 10). In the cELS however, ENTPD3 was the second highest protein expressed. The ENPP1 levels were significantly lower than the levels of ENTPD3 (*p* < 0.0001), and did not differ from ENTPD2 (*P* = 0.6412), ENTPD8 (*P* = 0.9639), NT5E (*P* = 0.9431) or TNAP (*P* = 0.9657), 2way ANOVA with Tuckey multiple comparisons test (Figure 11). In the urothelium homogenates, the ENPP1 levels were higher than the ENPP3 levels (*p* < 0.0001) (Figure 10) whereas in the cELS, the ENPP1 levels were significantly lower than the ENPP3 levels (*P* = 0.0006).

## 4 Discussion

The present study reports two major findings: 1) distention of the bladder wall during filling is associated with increased metabolism of extracellular purine nucleotides in the LP, and 2) multiple soluble enzymes contribute to mechanosensitive degradation of purines in the LP, in addition to membrane-bound nucleotidases. To the best of our knowledge, mechanosensitive degradation of extracellular mediators has not been reported previously. Such mechanisms in the LP likely contribute to guaranteeing a proper ratio of excitatory-to-inhibitory purine mediators deep in the bladder wall that is necessary to maintain adequate bladder excitability during bladder filling.

During filling, the volume of the urine increases dramatically while the intravesical pressure increases modestly until a point is reached where pressure rises precipitously and voiding occurs. There is no definite explanation of how the urothelium communicates with other cells in the bladder wall to maintain continence or initiate voiding. As discussed in Introduction, based on numerous observations in bladder mucosa sheets, cultured urothelial cells, or in the bladder lumen, a prominent role for urothelial ATP has been proposed in bladder mechanosensation, and mechanotransduction. However, there is still incomplete understanding of the physiological roles of ATP released from the bladder mucosa at low and high intravesical volumes and pressures (Mutafova-Yambolieva and Durnin, 2014; Takezawa et al., 2016b; Dalghi et al., 2020). Studies have suggested that afferent neuron activity and the micturition reflex are attenuated in bladders of mice lacking P2X_2_, P2X_3,_ and P2X_2,3_ purinergic receptors (Cockayne et al., 2000; Vlaskovska et al., 2001). Recent studies using newer technologies, however, demonstrated unaltered micturition reflex in *P2rx2* ^*−/−*^ and *P2rx3* ^*−/−*^ mice, calling into question the roles of ATP and P2X_2/3_ receptors in the normal micturition reflex (Takezawa et al., 2016a). It is generally assumed that ATP is released from urothelial cells in response to elevated hydrostatic pressure (Ferguson et al., 1997; Dunton et al., 2018). Some reports, however, have argued that stretch and not hydrostatic pressure induces release of ATP from the bladder mucosa (Yu, 2015). Importance of extracellular purinergic signaling in bladder physiology and pathophysiology is widely acknowledged (Merrill et al., 2016; Andersson et al., 2018). Therefore, controversies in the field require further studies to elucidate the role of ATP and other purines in the regulation of bladder excitability during authentic bladder filling.

In the present study, we used a bladder preparation devoid of the detrusor smooth muscle layer to obtain direct access to the LP surface. We demonstrated previously that the volume-pressure relationships of bladder preparations with and without the detrusor layer of muscle were similar (Durnin et al., 2019b), suggesting that the model is suitable for studying mechanisms during authentic bladder filling. We demonstrated that the LP layer of the preparation is preserved when the detrusor layer is carefully removed by cutting (not “peeling”) it away from the urothelium (Durnin et al., 2019b). The model was instrumental in confirming that ATP is released in the anti-luminal side of the urothelium at rest and during bladder filling and in suggesting that observations made at the luminal side of bladder mucosa do not reflect faithfully mechanisms in the LP. A surprising finding of the study was that the distribution of adenine purines in the LP at the end of bladder filling was ADO >> AMP > ADP >> ATP so that ATP represented only 5% of the total purines (Durnin et al., 2019b). It is logical to assume that this distribution of purines is primarily determined by sequential hydrolysis of ATP that was released at the LP during bladder filling. Since ATP release on the anti-luminal side of the urothelium is assumed to be caused by mechanical stretch, the goal of the present study was to determine whether the ATP degradation in the LP also changes with distention/stretch of the bladder wall during filling. Thus, we centered the study on metabolism of ATP in the LP of nondistended and distended denuded bladder preparations.

As in previous studies (Todorov et al., 1997; Durnin et al., 2012), we used 1,*N* ^*6*^-etheno-derivatives of ATP, ADP, and AMP as substrates. The enzymatic activities were evaluated by measuring the decrease of substrates and the increase of products in the LP of nondistended and distended preparations. Use of etheno-purines instead of authentic purines as substrates provides a number of advantages: 1) ecto-nucleotidases process 1,*N* ^6^-etheno-bridged purine nucleotides similarly to their endogenous counterparts (Jackson et al., 2020b), 2) the sensitivity of detection of etheno-nucleotides and nucleosides is 1,000,000-fold greater than the sensitivity of most detection methods for authentic purine nucleotides (Bobalova et al., 2002); this allows detection of small changes in substrate and product concentrations; 3) unlike authentic AMP and ADO, eAMP, and eADO cannot be diverted to other metabolic pathways *via* deamination because the etheno bridge blocks the *N* ^6^ nitrogen in the AMP and ADO molecules; this simplifies to some extent data interpretation; and 4) possible release of endogenous adenine purines remains undetected since the samples that were in contact with tissue do not undergo further etheno-derivatization. To examine the degradation of purine substrates at physiological levels, we chose a concentration (i.e., 2 µM) that is analogous to the concentration of purines previously determined in the same bladder model and with the same detection methodology (Durnin et al., 2019b). Monitoring the enzymatic activities for 1 hour following addition of substrate to the chamber with the bladder preparation seemed to be physiologically relevant as normal mice void on average 3–4 times within a 4-h period (Chen et al., 2017; Marshall et al., 2020). As anticipated, eATP was degraded to eADP, eAMP, and eADO in contact with the LP of nondistended detrusor-free bladder preparations so that eATP was diminished by 50% 1 h after initiation of reaction. A surprising observation was that eATP was decreased significantly more (by about 80%) in distended preparations for the same time period (i.e., 1 h) after initiation of the enzymatic reaction. This is a particularly intriguing observation, suggesting that during distention of LP caused by bladder filling, the degradation of ATP to ADP, AMP, and ADO is significantly enhanced. Similarly, the degradation of eADP in the LP of distended preparations exceeded the degradation of eADP in nondistended preparations. Interestingly, the degradation of eAMP in the LP was similar in distended and nondistended preparations. These data suggest that nucleotidases with different sensitivities to stretch appear to be present in the LP. The relative extent of substrate catabolism in the course of 1 h was eATP > eADP > eAMP.

We next asked whether eATP hydrolysis occurs in the suburothelium in the presence of detrusor. Microdialysis has been instrumental in examining release of small-molecule substances in the interstitial space of brain, skeletal muscle, liver, kidney, adipose tissue, and skin (Thompson and Shippenberg, 2001; La Favor and Burnett, 2016; Jackson et al., 2020b), but has not been utilized in studies of the bladder wall. In proof-of-principle experiments, we applied eATP *via* a MD probe inserted between the urothelium and the detrusor of *ex vivo* bladder preparations. Despite the extremely limited surface of the microdialysis membrane (∼0.6 mm^2^), the very low internal volume of the MD probe at the membrane (∼0.1 µl), and the short contact of the eATP substrate with LP (∼6 s), eATP produced eADP, eAMP, and eADO when perfused through the MD probe. Because the eATP breakdown occurred in such a small fraction of the LP for such a short contact between substrate and enzymes, distention-dependent eATP hydrolysis could not be revealed in these experiments. Importantly, however, this study provided affirmation that eATP can be degraded in the suburothelium and that results obtained in detrusor-free preparations can be extrapolated to the multilayer bladder wall.

Enhanced ATP degradation during filling explains why ATP represents such a small portion of the total purine pool in the LP at the end of bladder filling (Durnin et al., 2019a). However, this observation is at odds with the idea that the concentration of ATP that is released from the urothelium into the LP must reach maximum levels at the end of bladder filling to activate afferent neurons in the LP and trigger the micturition reflex (Burnstock, 2014). Factors other than ATP may be more important for initiation of voiding at the end of bladder filling. Moreover, accumulated ADO in the LP at end of filling might be necessary for optimal bladder excitability and can prevent overactivation of afferent neurons and other neighboring types of cells by ATP, including detrusor smooth muscle cells.

Several mechanisms could underlie increased degradation of purines in preparations that were distended by filling with physiological solution. One possibility is that biaxial stretch of the urothelium during bladder filling is accompanied by increase of surface area, which results in better access of substrate to membrane-bound enzymes. Another possibility is that stretch of cells in the urothelium transduce the mechanical signal into a cascade of biochemical signals resulting in actin cytoskeleton rearrangement, which in turn alters cell shape and plasma membrane assembling (Kessels and Qualmann, 2021). Although future studies are warranted to define such mechanisms, they could not explain straightforwardly the substrate specificity we observed. A third possibility is that increased degradation of purines during stretch is due to additional involvement of soluble enzymes that are released from the anti-luminal surface of the urothelium into the LP. Indeed, we found that eATP and eADP were hydrolyzed to their products when added to solutions that were previously in contact with the LP of denuded bladders. Once again, the degradation of purine substrates in ELS from distended preparations exceeded the degradation of purines in ELS from nondistended preparations. We concluded, therefore, that spontaneous and distention-induced release of nucleotidases occur in the LP. Such soluble nucleotidases, in addition to membrane-bound enzymes, likely control the ultimate availability of bioactive purine mediators in the vicinity of specific purinergic receptors in the LP during bladder filling.

Four major enzyme families are involved in extracellular purine metabolism: 1) the ENTPD family (EC 3.6.1.5), 2) the ENPP family (EC 3.6.1.9; EC 3.1.4.1), 3) Ecto-5-nucleotidase NT5E/CD73 (EC 3.1.3.5), and 4) Alkaline phosphatases (EC 3.1.3.1). (Zimmermann et al., 2012; Yegutkin, 2014). ENTPD1,2,3, and 8 are cell surface-located enzymes that hydrolase preferentially extracellular ATP and ADP whereas ENTPD4–7 are localized in intracellular organelles and have low affinity for ATP (Zimmermann et al., 2012). Of the ENPP family, ENPP1 displays highly efficient ATP hydrolysis to generate AMP and inorganic pyrophosphate PPi. ENPP3 also hydrolases ATP, but to a lesser degree than ENPP1. ENPP4 has been shown to degrade ATP *in vitro*, but the ATP hydrolysis rate by ENPP4 is negligible compared to that of ENPP1 (Borza et al., 2021). Purine nucleotides are not preferred substrates for ENPP2,5,6, and 7 as these enzymes have evolved as phospholipases with phosphodiesterase activities (Borza et al., 2021). Mammalian alkaline phosphatases (ALPLs) are expressed ubiquitously in multiple tissues, display broad substrate specificity and can hydrolyze ATP, ADP, AMP, and PPi among other substrates (Zimmermann, 2006). In fact, ALPLs are the only ecto-nucleotidases that can sequentially dephosphorylate nucleoside triphosphates to the nucleoside. Three isozymes are tissue specific with highly restricted expression to the placenta, germ cells, and intestines, while the fourth isozyme, tissue non-specific alkaline phosphatase (TNAP) is present in numerous tissues, including the kidney, liver, bones, and the central nervous system (Sebastián-Serrano et al., 2014). It is presently unknown whether TNAP plays a role in establishing the ATP/ADO ratio in the bladder wall. NT5E/CD73 is another integral component of the purinergic system. NT5E/CD73 is a ubiquitously expressed glycosylphosphatidylinositol-anchored glycoprotein (GPI-AP) that catalyzes the last step in the extracellular metabolism of ATP to form ADO. Members of the ENTPD and ENPP families generate AMP from ATP and ADP. Subsequent hydrolysis of AMP to ADO is primarily, but not exclusively, carried out by NT5E/CD73 (Alcedo et al., 2021). In summary, current knowledge about membrane-bound nucleotidases suggests that ENTPD1,2,3, and 8, ENPP1 and 3, NT5E/CD73, and ALPL/TNAP are of most significance to the extracellular hydrolysis of ATP and ADP.

The information about distribution, localization and function of nucleotidases in the non-disease bladder is rather limited. Immunohistochemistry studies of the mouse urinary bladder (Yu et al., 2011; Babou Kammoe et al., 2021) reported that ENTPD1 was expressed primarily on the surface of detrusor smooth muscle cells and in the LP, ENTPD2 was localized between smooth muscle bundles and in the LP, ENTPD3 and 8 were localized in the urothelium and were not resolved in the detrusor layer. NT5E was found localized in detrusor (Yu et al., 2011; Babou Kammoe et al., 2021) and to a lesser degree in the LP (Babou Kammoe et al., 2021). In another immunohistochemistry study, ENTPD3 and ALPL appeared to be localized on basal and intermediate cells of the urothelium but not on umbrella cells (Yu, 2015), suggesting asymmetrical distribution of nucleotidases through layers of the bladder mucosa. The impact of these enzymes on bladder excitability could be profound as they have the potential to inactivate agonists of P2X and P2Y purinergic receptors and to produce ADO as an agonist of the four ADO receptors. In addition, production of the nucleoside ADO secures purine salvage through cellular reuptake of the nucleoside *via* equilibrative nucleoside transporters and re-phosphorylation to AMP inside the cell.

Members of the nucleotidase families demonstrate relative substrate specificity for adenine purines. ENTPD1, 3, and 8 hydrolyze both ATP and ADP; however, the hydrolysis of ATP is more rapid than the hydrolysis of ADP. ENTPD1 hydrolyzes ATP directly to AMP with minimal formation of ADP as an intermediate product (Kukulski et al., 2005; Knowles, 2011). The appearance of ADP could be demonstrated upon hydrolysis of ATP by ENTPD3 and 8 (Kukulski et al., 2005). ENTPD2 hydrolyses primarily ATP to ADP, but it does not hydrolyze ADP causing considerable accumulation of ADP before it is further hydrolyzed to AMP (Zimmermann et al., 2012). ENPP1 preferentially hydrolyses ATP, but it also hydrolyses other nucleotides including ADP (Kumar and Lowery, 2021). As aforementioned, NT5E is the main enzyme that converts AMP into ADO. Importantly, ATP and ADP are competitive inhibitors of mammalian NT5E with Ki values in the low micromolar range (Sträter, 2006). As a result, when cells release ATP or ADP, NT5E is inhibited until ATP and ADP are mostly metabolized to AMP (Zimmermann et al., 2012). This “feed-forward” inhibition could explain the delayed formation of eADO from eATP or eADP in the present study. Likewise, accumulation of ATP and ADP in the presence of ENTPD inhibitors would diminish the ADO production due to inhibited breakdown of AMP by NT5E. Since TNAP can metabolize ATP all the way to ADO, this enzyme may provide an alternative pathway for ADO production, if present.

While membrane-bound forms of nucleotidases have been studied extensively, soluble forms of nucleotidases have received rather little attention. Some amounts of ENTPDs, ENPPs, CD73, and alkaline phosphatase appear to constitutively circulate in human bloodstream and their levels increase in disease states such as inflammation and cancer (Yegutkin et al., 2007; Zimmermann et al., 2012; Borza et al., 2021). Neuronal release of nucleotidases has been suggested as a mechanism for neurotransmitter inactivation (Todorov et al., 1997). In any event, soluble forms considerably broaden the reach of the enzymes by diffusion within a tissue or by their distribution within tissue fluids. To the best of our knowledge, no data are yet available on regulation of bladder excitability by enzymes that are released from the urothelium in the course of bladder filling. One hour after initiation of reaction in large-volume ELS (i.e., 2.5-ml) removed from the tissue bath, eATP was diminished by 20 and 40% in nondistended and distended preparations, respectively, suggesting that eATP-degrading enzymes were released in a stretch dependent manner. Under the same experimental conditions, only 10%–15% of eADP was degraded by released enzymes. The action of released enzymes was significantly underestimated in these studies, because the reactions were carried out in a 500-fold higher volume than the volume of LP which is about 50 µm thick in the mouse bladder (Winder et al., 2014). In 12.5-fold concentrated ELS, eATP was reduced by ∼50 and ∼80% in nondistended and distended preparations, respectively. The eADP substrate decreased by ∼10 and ∼30% in cELS from nondistended and distended bladders, respectively. Clearly, distention of the urothelium and suburothelium/LP is associated with greater catabolism of ATP and ADP by soluble nucleotidases that are released in the LP during bladder filling.

All plasma membrane nucleotidases require millimolar concentrations of divalent cations Mg^2+^ and Ca^2+^ for maximal activity (Kukulski et al., 2005; Zimmermann et al., 2012). As part of the general characterization of released enzymes, we evaluated the relative importance of the two cations for the activity of released enzymes. According to MAXCHELATOR [WEBMAXC EXTENDED (ucdavis.edu)] (Bers et al., 2010), 5 mM EGTA chelates ∼99.99% of Ca^2+^ and only ∼8% of Mg^2+^ in regular KBS (pH 7.4, 37°C) whereas in the absence of Ca^2+^, ∼12% of Mg^2+^ is chelated by 5 mM EGTA. In contrast, 5 mM EDTA chelates ∼99.99% of both cations under the same conditions. We took advantage of the different chelating properties of EGTA and EDTA and measured the degradation of eATP and eADP in solutions that contained released enzymes but lacked Ca^2+^ and/or Mg^2+^ with or without addition of EGTA or EDTA. As anticipated, removal of both Ca^2+^ and Mg^2+^ abolished the hydrolysis of eATP and eADP, indicating that Ca^2+^ and Mg^2+^ are crucial for optimal activities of soluble “ATPases” and “ADPases” in the LP. The presence of Ca^2+^ alone or Mg^2+^ alone appeared to be sufficient for the ability of soluble “ATPases” to hydrolyze ATP. Surprisingly, the hydrolysis of eATP was enhanced in the presence of Mg^2+^ and absence of Ca^2+^. It is possible that Ca^2+^ and Mg^2+^ “compete” for maintaining optimal activity of soluble enzymes so that when Ca^2+^ is removed, Mg^2+^ takes over the maintenance unrestrained. The formation of eADP from eATP followed the relationships described for the eATP decrease: no change in the absence of Mg^2+^ alone, enhanced formation of eADP in the absence of Ca^2+^ plus EGTA, and abolished eADP formation when both Ca^2+^ and Mg^2+^ were absent. Despite increased formation of eADP in the absence of Ca^2+^, neither the formation of eAMP nor of eADO were significantly enhanced. In fact, the hydrolysis of eADP was modestly inhibited in the absence of Mg^2+^ and significantly inhibited in the absence of Ca^2+^, suggesting that soluble ADPase(s) requires Ca^2+^ more than Mg^2+^ for its activity. This is in contrast with what we observed with the eATP hydrolysis. Altogether, the present study establishes that Ca^2+^ and Mg^2+^ are essential for the activities of soluble nucleotidases that are released in the LP during bladder filling. However, releasable enzymes that degrade sequentially ATP to ADO appear to depend unequally on extracellular Ca^2+^ and Mg^2+^: thus, Mg^2+^ might compete with Ca^2+^ for ensuring optimal activity of enzymes that convert ATP to ADP, whereas Ca^2+^ may be more important for the activity of nucleotidases that catabolize ADP to AMP and then to ADO.

We next examined how activities of soluble enzymes in the LP are affected by commonly-used inhibitors of membrane-bound nucleotidases. In cELS collected from nondistended or distended preparations, ARL67156 modestly diminished the decrease of eATP, significantly reduced the increase of eAMP and eADO, but did not affect the intermediate product eADP, suggesting different sensitivity of soluble nucleotidases to ARL67156. A study using recombinant mouse nucleotidases has demonstrated that ARL67156 is a weak competitive inhibitor of ENTPD1, ENTPD3, and ENPP1, and is not an effective inhibitor of ENTPD2 and ENPP3, and inhibits the mouse ENTPD8 (Levesque et al., 2007). POM-1 was proposed as a potent inhibitor of membrane-bound ENTPD1–3 (Muller et al., 2006). In the present study, POM-1 was a more potent inhibitor of the degradation of eATP than ARL67156 and significantly reduced the formation of all three products. The discrepancy in the effects of POM-1 and ARL67156 on eATP hydrolysis was likely due to different efficacy toward the activity of ENTPD2. POM-1 inhibits ENTPD2 (Muller et al., 2006) whereas ARL67156 does not (Levesque et al., 2007). ENTPD2 hydrolyses ATP to ADP with minimal AMP accumulation (Zimmermann et al., 2012). The effects of ARL67156 in the present study suggest that soluble enzymes similar to ENTPD1 and 3, and ENPP1 might be released in the LP whereas the effects of POM-1 suggest that an ENTPD2-like enzyme could also be released in LP during bladder filling. While a number of non-selective ENTPD inhibitors have been developed, potent subtype-specific inhibitors are scarce. PSB06126 is an ENTPD3 inhibitor of the rat isoform of the enzyme and appears to display selectivity over ENTPD1 and 2 (Baqi et al., 2009). It may have similar efficiency profile in the mouse since mouse and rat ENTPD3 share 96% homology (UniProt). In ELS collected from distended preparations, PSB06126 diminished the eATP decrease and the eADP increase and had no effect on eAMP and eADO increase. The ENPP1 Inhibitor C (Kawaguchi et al., 2019) diminished the decrease of eATP but did not affect the formation of eADP since ENPP1 hydrolyses eATP to eAMP directly. Consequently, the formation of eAMP was diminished by ENPP1 inhibitor C. Neither PSB06126 nor ENPP1 Inhibitor C had an effect on the degradation of eATP in nondistended preparations. The TNAP inhibitor L-p-BT (Jackson et al., 2020a) had no effect on eATP decrease or product increase in nondistended or distended preparations. Together, these results suggest that no spontaneous release of ENTPD3, ENPP1, and TNAP occurred in the LP whereas distention of the LP during bladder filling likely released ENTPD3-and ENPP1-like enzymes, but not TNAP. The relative potency order of nucleotidase inhibitors for distention-released soluble nucleotidases in LP was POM-1 > ARL67156 > PSB06126 = ENPP1 inhibitor C. The pharmacological characterization suggests a release of cocktail of nucleotidases in the LP during bladder filling that likely include ENTPD1–3, ENPP1 and ENPP3, and possibly ENTPD8. However, no indication of the presence of TNAP was obtained with the use of a pharmacological inhibitor.

Poor specificity of enzyme inhibitors poses serious limitations for determining the identity of released enzymes by pharmacological characterization. Therefore, we next sought to determine whether the enzymes suggested by pharmacology studies are indeed present in cELS from distended bladder preparations. Using a highly-sensitive methodology for protein detection was crucial for these experiments given the high dilution of released enzymes in the EL samples. Therefore, we used a capillary western blot method that has the advantage of being a more sensitive and automated approach for protein detection while providing comparable results to traditional western blotting (Chen et al., 2013; Lu et al., 2018). Multiple nucleotidases were found present in urothelium homogenate and in cELS. Of particular interest is the observation that the relative expression of enzymes released in cELS was different from the relative expression of nucleotidases in the mouse urothelium, suggesting that a regulated release of enzymes likely occurs in the LP during bladder filling. Thus, the relative distribution of nucleotidases in urothelium was ENTPD1>>ENPP1>ENTPD2 = ENTPD3 > ENPP3 = NT5E >> ENTPD8 = TNAP and ENTPD1>>ENTPD3>>ENPP3> ENPP1 = ENTPD2 = NT5E >> ENTPD8 = TNAP in cELS. The findings that several enzymes with nucleotidase activities were present in cELS are in accordance with the results from substrate degradation and pharmacological characterization of soluble enzymes in cELS. An intriguing observation was that enzymes that have transmembrane domains in their molecular structure were found released in the solutions bathing the LP. A possible mechanism underlying such release could be through proteolytic removal of membrane protein ectodomains (aka ectodomain shedding) that is a post-translational modification regulating functions of hundreds of membrane proteins (Lichtenthaler et al., 2018). The cleavage reactions are catalyzed by a broad range of proteases, including intramembrane and soluble proteases that can cleave single-pass transmembrane proteins, dual-pass and polytopic membrane proteins or proteins that are attached to the external leaflet of the plasma membrane by a GPI anchor (Lichtenthaler et al., 2018). As discussed, soluble forms of nucleotidases have been reported (Jiang et al., 2014). For example, NT5E and TNAP are GPI-anchored proteins that can be released by endogenous phospholipase C cleavage of GPI, *via* microvesicles or *via* exosomes from cancer cells (Zimmermann et al., 2012). ENPP1 is a single-pass transmembrane enzyme (Borza et al., 2021) whereas ENTPD1,2,3, and 8 have two transmembrane domains close to their N- and C-termini and a large extracellular loop that contains the catalytic domain (Zimmermann et al., 2012). ENTPD1 is preferentially targeted to caveolae, membrane microdomains with specialized structure and function (Kittel et al., 1999; Koziak et al., 2000). There is evidence that catalytically active ENTPD1 can be shed in membrane-bound form from plasma membranes of ENTPD1-expressing cells (Yegutkin et al., 2000; Ceruti et al., 2011) or to be incorporated in membrane particles that are released in the extracellular space (Barat et al., 2007). Shedding of undefined ATP-degrading enzymes has been observed from endothelial cells in response to shear stress and from cultured astrocytes following O_2_-glucose deprivation (Yegutkin et al., 2000; Ceruti et al., 2011). Studies have suggested that mechanical stretch could enhance the expression of enzymes involved in steroid biosynthesis (Feng et al., 2019). To the best of our knowledge, however, release of membrane-bound enzymes in response to stretch has not been reported previously. In all membrane-bound nucleotidases, the transmembrane and intracellular domains comprise approximately 10%–13% of the molecule (UniProt), suggesting that no significant differences in the mass of membrane-bound and cleaved enzymes would be detected with western immunoblot methodologies. This might explain the similar masses (molecular weights) of enzymes that are present in urothelial tissue homogenates and in cELS. Studies have suggested that lack of transmembrane domains of nucleotidases may result in reduced catalytic activity (Wang et al., 1998; Grinthal and Guidotti, 2002). Soluble forms of human ENTPDs engineered to comprise of only the extracellular domains demonstrated markedly diminished nucleotide-hydrolyzing capabilities in comparison with native membrane-embedded ENTPDases containing two transmembrane domains (Knowles, 2011). It is currently unknown whether this is true for the enzymes released in the LP during bladder filling. As demonstrated, however, the degradation of eATP by such enzymes was distinct.

There are two important implications of the present study: 1) multiple enzymes that coexist on the cell surface or are released during bladder filling determine the effective concentrations of biologically active mediators in the LP and the degree of activation of purinergic receptors in various types of cells in the bladder wall, and 2) in studies of purinergic signaling, it is important to investigate simultaneously the availability of extracellular ATP and its metabolites.

**FIGURE 12:**
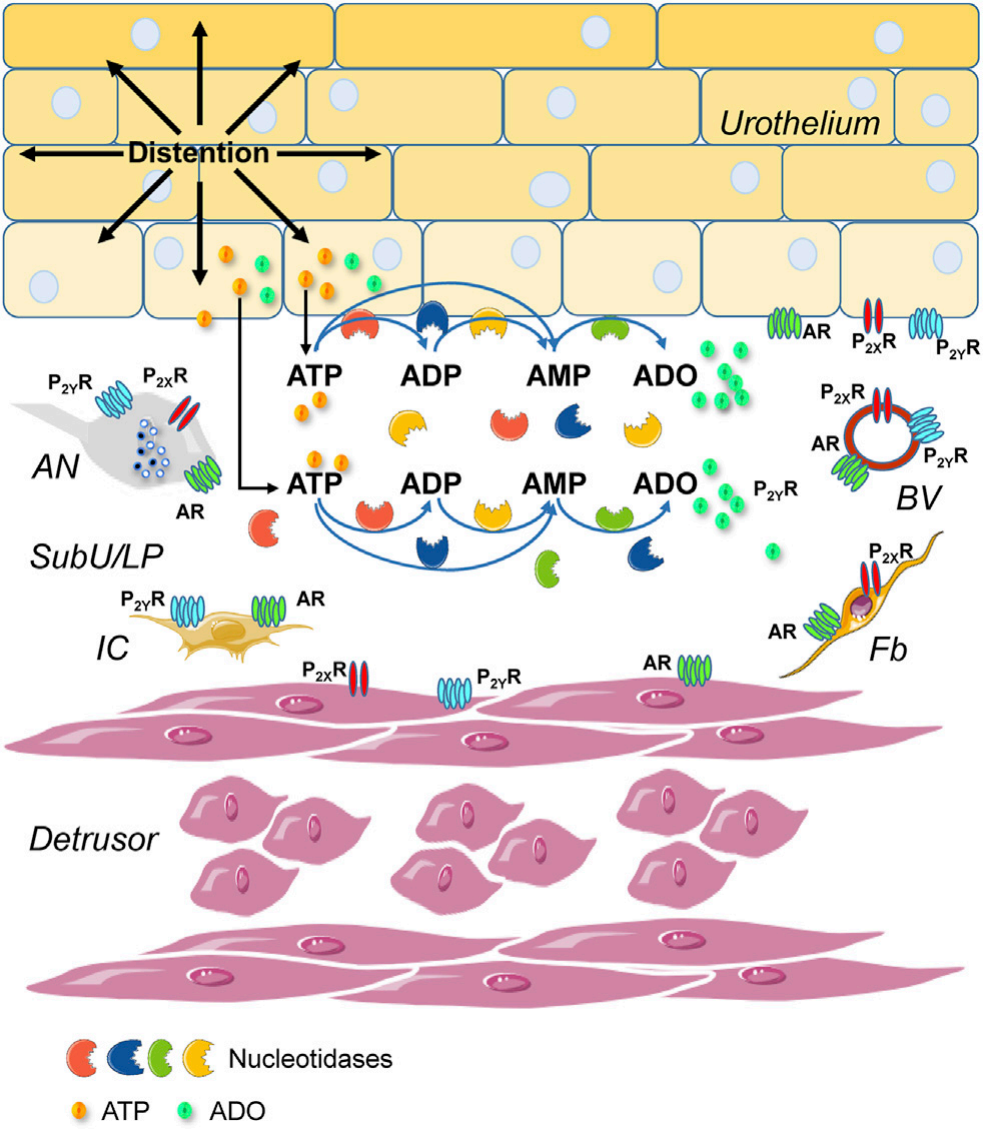
A model depicting mechanisms of purinergic signaling in the lamina propria during bladder filling. Stretch of the bladder wall during filling causes release of ATP from the urothelium into the suburothelium (Birder and Andersson, 2013; Burnstock, 2014; Dalghi et al., 2020). ATP activates P2X (e.g., P2X2/X3) receptors (P2XR) on afferent nerve terminals in urothelium and suburothelium/lamina propria (SubU/LP) and triggers a voiding reflex (Cockayne et al., 2000; Vlaskovska et al., 2001). ATP that is released in the LP is hydrolyzed to ADP, AMP, and adenosine (ADO) by four families of membrane-bound nucleotidases (Zimmermann et al., 2012). Bladder excitability during filling is regulated by excitatory (ATP and ADP) and inhibitory (ADO) purine mediators in the LP that activate specific purinergic receptors. ATP activates ligand-gated P2XR and G protein-coupled P2Y receptors (P2YR), ADP activates P2YR, and ADO activates G-protein coupled adenosine receptors (AR) (Burnstock, 2014). P2XR, P2YR, and AR are ubiquitously expressed in the bladder wall, including in cells in the detrusor, the urothelium, and the LP (Dalghi et al., 2020). Cell types that express purinergic receptors in the LP include afferent neurons (AN), interstitial cells (IC), fibroblasts (Fb), and blood vessels (BV). Nucleotidases have the ability to terminate P2XR or P2YR responses initiated by ATP and to favor the activation of AR or ADP-responding receptors. In addition to the membrane-bound nucleotidases, enzymes that metabolize ATP are released in the LP spontaneously and during distention of the bladder wall during bladder filling. Released enzymes degrade ATP to ADP, AMP, and ADO. The activity of released enzymes is greater in distended LP than in nondistended LP indicating mechanosensitive release of enzymes. Soluble nucleotidases in the LP identify with several membrane-bound nucleotidases and are possibly released in the LP by distention-induced ectodomain shedding (Lichtenthaler et al., 2018). Distention-dependent degradation of ATP by membrane-bound and soluble nucleotidases diminishes the presence of ATP in the LP at the end of bladder filling (Durnin et al., 2019b) to prevent abnormal excitability of the bladder. The proper availability of excitatory and inhibitory purines in the bladder wall is determined by distention-associated purine release and purine metabolism.

In summary (Figure 12), here we report that several soluble nucleotidases are released in the LP during bladder filling causing enhanced hydrolysis of ATP and ADP at the end of filling. These findings corroborate the hypothesis that distention induced-release of soluble nucleotidases attenuates the excitatory effects of ATP and ADP on cells in the LP and in detrusor and suggest the prospect that inhibition of purine nucleotide degradation could aggravate bladder excitability by accumulation of excitatory urothelial mediators in the bladder wall.

At present, we do not know the mechanisms underlying the distention-induced enzyme release in the LP. The mechanisms of coordination of multiple enzyme activities in regulation of bladder excitability also remain to be unraveled. Future studies aimed at understanding the complexity of purinergic regulation and functions in the bladder will help to guide translational and clinical efforts for bladder motility disorders, inflammation, cancer, and other human diseases.

## Data Availability

The raw data supporting the conclusion of this article will be made available by the authors, without undue reservation.

## Abbreviations

ADO, adenosine; ADP, adenosine 5′-diphosphate; ALPL, alkaline phosphatase; AMP, adenosine 5′-monophosphate; ATP, adenosine 5′-triphosphate; cELS, concentrated extraluminal solution; DMSO, dimethyl sulfoxide; EL, extraluminal; ELS, extraluminal solutions; ENPPs, ectonucleotide pyrophosphatase/phosphodiesterases; ENTPDs, ectonucleoside triphosphate diphosphohydrolases; GPI, glycosylphosphatidylinositol; GPI-AP, GPI-anchored protein; HPLC-FLD, high-performance liquid chromatography with fluorescence detection; KBS, Krebs solution; LP, lamina propria; LDH, lactate dehydrogenase; mKBS, modified Krebs-bicarbonate solution; NT5E, 5′-nucleotidase; TNAP, tissue nonspecific alkaline phosphatase.
